# The Dual Process model: the effect of cognitive load on the ascription of intentionality

**DOI:** 10.3389/fpsyg.2025.1451590

**Published:** 2025-03-07

**Authors:** Micaela Maria Zucchelli, Nicola Matteucci Armandi Avogli Trotti, Andrea Pavan, Laura Piccardi, Raffaella Nori

**Affiliations:** ^1^Department of Psychology, University of Bologna, Bologna, Italy; ^2^Department of Psychology, “Sapienza” University of Rome, Rome, Italy; ^3^San Raffaele Cassino Hospital, Cassino, Italy

**Keywords:** Dual Process model, Knobe effect, cognitive load, intentionality, side effect

## Abstract

**Background:**

The classic Dual Process model posits that decision-making is determined by the interplay of an intuitive System 1 and a logical System 2. In contrast, the revised model suggests that intuition can also be logical. The Cognitive load paradigm has been used to distinguish underlying rational and intuitive processes, as it tends to lead to the use of heuristics over reasoning. Through two studies, we aimed to investigate the impact of two increasing levels of extraneous cognitive load on intentionality decision-making by comparing the two decision-making models.

**Methods:**

The task required participants to attribute intentionality to negative and positive side effects, which were foreseeable but not deliberately intended. This compared an intuitive response, focused on the outcome, with a logical one, focused on the absence of intention. Participants were randomly assigned to one of the six experimental conditions, each with varying cognitive loads (high cognitive load vs. low cognitive load vs. no cognitive load) and scenario valence (negative vs. positive). In Study 1, cognitive load was made by introducing a concurrent task during intentionality attribution, whereas in Study 2, cognitive load was accomplished by associating the dual-task with a time pressure paradigm. Participants under cognitive load were expected to exhibit cognitive resource exhaustion, providing greater judgments of intentionality for negative outcomes and lower for positive ones, due to the dominance of intuition, compared to evaluations provided by participants who were not under cognitive load.

**Results:**

In both studies, cognitive load reduced intentionality attributions for positive side effects compared to the no-load condition, with response times being longer for positive side effects than for negative ones.

**Conclusion:**

This pattern suggests System 2 intervention for positive outcomes and System 1 dominance for negative ones. Therefore, introducing cognitive load enabled us to identify the different roles of the two decision systems in intentionality attribution.

## 1 Introduction

Making decisions is a crucial and essential ability in everyday life (Mather, 2006). Various disciplines, including philosophy and juror psychology (Phelps et al., 2014), have endeavored to elucidate the processes involved in decision-making (e.g., De Neys and Bialek, 2017; Morelli et al., 2022). Effective decision-making involves assessing various alternatives and choosing the best one according to individual capabilities, values, priorities, and convictions to achieve specific goals (e.g., Broche-Pérez et al., 2016; Dewberry et al., 2013; Reyna and Lloyd, 2006). Doya (2008) outlines four stages of the decision-making process: (a) recognize the present situation, assess potential actions and identify internal and external factors influencing them (i.e., the positive or negative outcome valence, likelihood, timeframe - short or long-term, cognitive workload, and the context); (b) option evaluation, in terms of reward or punishment; (c) select the action; (d) re-evaluate the action based on feedback received. Understanding human interactions from a third-person perspective and moral judgments is a key part of decision-making (e.g., Tinghög et al., 2016). Human moral judgments are largely shaped by the integration of two factors: the assessment of an agent’s causal responsibility for harm and the assessment of intent to cause harm by him/her (e.g., Martin et al., 2021; Malle et al., 2014; Cushman et al., 2013). According to a study conducted by Timmons and Byrne (2019), determining whether a moral violation is permissible or not is not always a straightforward task. When people are mentally overloaded, they tend to see a morally wrong action with a positive outcome (like saving five lives at the cost of one) as less acceptable. This happens because they usually create a simple mental model where the harmful action is not directly linked to its beneficial consequence. In contrast, the phenomenon does not occur when participants are not under cognitive load. Importantly, recent research suggests that when adults make moral judgments while experiencing cognitive load, they tend to exhibit a more childlike response pattern (Buon et al., 2013). As children grow older, their moral judgments transition from primarily considering the outcome, to emphasizing the actor’s intention to act (Margoni and Surian, 2016). Martin et al. (2021) found that in adults, cognitive load affects moral judgment, making it more focused on causation than intentions, specifically for accidental harm, which is judged more harshly despite the lack of malicious intention. In contrast, cognitive load does not affect judgments of attempted harm with malicious intent but no actual harm. This effect only influences the moral wrongness of an action, not the deserved punishment for the person responsible. Therefore, cognitive load plays a vital role in fostering intuitive thinking and assessing its impact on the mental representation of an event. Indeed, cognitive load can severely affect decision-making, leading individuals to resort to heuristics instead of controlled cognitive processes. They prioritize intuitive processing over slow processing if they already have a response (Evans and Stanovich, 2013; Kahneman, 2011). As a result, most people tend to rely on their initial response and overlook the logical consequences.

### 1.1 Dual Process models of thinking

According to the traditional Dual Process model of thinking (Evans and Stanovich, 2013; Evans, 2008; Kahneman, 2011; Sloman, 1996), decision-making is influenced by the interplay of two cognitive processes: the so-called System 1, which is automatic, intuitive, fast, experiential and affect-based and the System 2, which, on the other hand, is controlled, analytical, slow, deliberative and logical. Recently, De Neys and Pennycook (2019) have proposed a revised version of the Dual Process model, known as Dual Process Model 2.0. The model suggests that there are various types of intuitions. Some are logical and reliable, while others are heuristic and less reliable. Sometimes, one type dominates, and other times, the opposite type prevails. For example, our gut feelings may influence our decision-making at first. Then, our logical thinking may kick in and lead us to make a logical response without further thinking. According to the model, when the activation strengths of the heuristic and logical intuitions are more similar, there is a higher likelihood that the dominant intuition will be overridden through deliberation, resulting in a slower but more logical response. If the override fails, the reasoner will provide a heuristic response. Any deliberate processing will be then used, in this scenario, to find a clear justification for the dominant heuristic intuition (i.e., rationalization) (Pennycook et al., 2015). Furthermore, according to the model, System 1 can generate logical and intuitive responses without needing System 2 for more thoughtful consideration. In this case, if System 1 works effortlessly and does not need cognitive resources, then conflict detection should not occur between the two types of responses, in terms of different response times, under cognitive load (De Neys and Bialek, 2017). Previous studies on intuition and moral decision-making have used cognitive load manipulations to uncover the mechanisms underlying moral judgments. When making moral decisions, there is, traditionally, a comparison between a utilitarian/deliberate response, such as the option to sacrifice one person in order to save many others, which has been associated with deliberate System 2 processing, and a deontological/intuitive response, which on the other hand, refuses to cause harm and has been associated with intuitive System 1 processing. The core idea is that giving a utilitarian response to moral dilemmas requires engaging in System 2 thinking, allocating cognitive resources to override an intuitive System 1 response, which primes us not to harm others (Greene et al., 2001; Greene et al., 2004; Greene, 2014; Paxton et al., 2012). Specifically, many studies using the cognitive load paradigm demonstrate that intuition leads to deontological/intuitive judgments in moral dilemmas (e.g., trolley dilemma; Greene et al., 2008; Moore et al., 2008; Paxton et al., 2012). Later studies have expanded this Dual Process framework to intent-based moral judgments, illustrating its broader applicability beyond traditional moral dilemmas (Buon et al., 2013, 2016; Cushman, 2008; Cushman et al., 2013). The hypothesized underlying mechanism is very similar to that originally proposed by Greene et al. (2001, 2004). It posits the existence of two competing responses: one that is faster, intuitive, and emotionally driven, focusing on the consequences of someone else’s action (and therefore on a causal explanation), and a slower one that involves reasoning and focuses primarily on the actor’s intentions (i.e., the mental state of the actor). Starting from the work of Cushman (2008), these two mechanisms have gained primary importance in evaluating cases where mental states and consequences of actions do not align, such as in scenarios of accidental or attempted harm. Later, Buon et al. (2016) proposed the ETIC model (E = Emotional arousal, T = Theory of mind, IC = Inhibitory control) to explain and account for the cognitive-affective process underlying moral judgment. This model highlights the integration of the causal role of the agent with their intentions when assessing harmful actions. Following this model, moral judgments depends on three key factors: (i) emotional arousal generated by evaluating the situation, (ii) theory of mind abilities for assessing the actor’s intention, (iii) and inhibitory control for down-regulating emotional arousal arising from incongruence between the actor’s intentions and the action’s consequences, such as in case of accidental harm. As the authors explain, “*the ETIC model is the first attempt to integrate and develop the important theoretical propositions of Greene and Cushman into a comprehensive account of the processes involved in our judgments of basic harmful actions*” (Buon et al., 2016, p.1663). Similarly, to the models proposed by Greene et al. (2004) and Cushman (2008), the ETIC model suggests that the core of morality lies in an emotional intuition triggered by the presence of a harmful situation. This intuition is subsequently regulated or inhibited by higher-order cognitive systems. However, proposing a more complex working interaction between inhibitory control abilities, theory of mind and emotional arousal. There are situations between intentional and accidental actions, such as recklessness or negligence, where judging intentionality becomes more complex. This paper focuses on one such situation, commonly referred to in the literature as the Knobe effect (Knobe, 2003).

### 1.2 The Knobe effect

The study aims to compare the classic Dual Process model with the Dual Process Model 2.0 to analyze the impact of the cognitive load paradigm on adults performing a specific task. This task encompasses two types of scenarios in which the actor, intentionally pursuing a specific goal, encounters a side effect that is predictable but not deliberately intended. In one scenario, the side effect leads to a positive outcome; in the other, it results in a negative outcome (referred to as the Knobe Effect, KE; Knobe, 2003). People tend to assume intentionality when a negative side effect occurs, but they do not when the side effect is positive (Knobe, 2003). In the first case, this intuitive response is not logical because the person did not intend to cause the side effect, while in the second case, the intuitive response— not attributing intentionality to the side effect—aligns with the logical explanation. This effect seems to be highly robust, as it has been replicated in several different scenarios (Mele and Cushman, 2007; Ngo et al., 2015), across different age groups (Leslie et al., 2006) and in individuals with various personality traits as well (Zucchelli et al., 2018). Several studies have attempted to understand and explain the discrepancy in attributing intentionality to positive and negative side effects. They have explored various aspects, including the role of prior moral judgment (e.g., Lee and Shapiro, 2014; Knobe, 2022), the emotional response to negative outcomes (e.g., Ngo et al., 2015; Zucchelli et al., 2019), and the actor’s mindset (e.g., for a review see Cova et al., 2016; Feltz, 2007).

The Knobe effect challenges the traditional view of intentional action, which holds that intentions precede moral judgments, by showing how, in these cases, perceived intentionality varies depending on the valence of the consequences and, consequently, the resulting moral evaluation (Laurent et al., 2021). As shown and hypothesized by many authors, intentionality attribution in the context of the KE is influenced by, and slightly a consequence, of moral judgment (Cova et al., 2016; Knobe, 2010; Laurent et al., 2021; Leslie et al., 2006; Pettit and Knobe, 2009). As stated above, prior research has investigated cognitive aspects of moral judgment manipulating intentionality (e.g., intentional vs. accidental or attempted harm). However, the Knobe effect manipulates the valence of consequences (negative vs. positive side effects) to examine whether this influences how intentionality is perceived. The Knobe effect illustrates that the perception of intentions can be shaped by various processes, such as moral evaluations, rather than being solely determined by an agent’s explicit intentions. When assessing a situation, the KE shows how the integrated combination of intention and consequences can significantly influence the shift between evaluating an action as more intentional or accidental (Shen et al., 2011; Margoni and Surian, 2022).

Our aim is to focus on the cognitive aspects to gain a deeper understanding of KE. Indeed, by examining the comparison between an intuitive response and a rational one, especially in the moral domain, KE emerges as an ideal field to explore the impact of cognitive load on a specific type of decision-making, intentionality attribution. To our knowledge, only one study has examined the impact of reasoning on mitigating this phenomenon. However, this study only focused on considering individual differences among people: Pinillos et al. (2011) combined the KE scenarios with the Cognitive Reflection Test (CRT- Frederick, 2005). The CRT is a task that measures a person’s inclination to override an initial incorrect response and engage in reflective thinking to arrive at the correct solution. The study revealed that individuals who excelled in the reasoning task, showcasing a greater inclination toward deliberation, were assigned lower intentionality judgments for the negative scenario. In the KE scenario, we have a foreseeable and unintended consequence. This situation can be challenging to interpret because the agent is aware of the consequences and is causing them, yet these consequences are not willed by the agent (Kirfel and Phillips, 2023). The valence of these consequences influences different kinds of intuitions, leading to different ascriptions of intentionality to the side effects. In the negative scenario, a negative side effect that could have been foreseen (and is acted upon anyway) generates a biased intuition. This is due to mechanisms such as emotional response and negative moral judgment, which lead to a strong tendency to attribute intentionality even when the action is clearly stated to be unintentional (Ngo et al., 2015; Zucchelli et al., 2019). According to the Dual Process Model 2.0, there is a strong heuristic intuition that “the side effect is intentional,” which often overrides the more logical intuition that “the side effect is foreseen but described as unintended and, therefore, not fully intentional.” In the positive scenario, the fact that someone does not aim to achieve a positive side effect, even if they can foresee it, creates a conflict (Why would not someone want a positive consequence?), leading to a tendency to deny intentionality for the positive outcome achieved (Knobe, 2022). In this case, the intuition aligns with the logical response described above: the side effect is foreseen but described as unintended and, therefore, not fully intentional.

### 1.3 Cognitive load and decision-making

Cognitive load refers to the amount of information a person’s working memory can effectively handle during task performance (Sweller, 1988). The Cognitive Load Theory (CLT; Sweller, 1988, 2010) proposes that cognitive load can be categorized into three types: (i) Intrinsic Cognitive Load, which refers to the complexity of the information being processed. This complexity is determined by the number of elements to consider simultaneously and their interconnections; (ii) Extraneous Cognitive Load, which refers to the challenges that arise when information is presented as less-than-optimal. This can result from inadequate instructional design, including unnecessary information or the separation of materials that should be integrated. We can refer to extraneous cognitive load as irrelevant and distracting information from the main task. These factors increase the demands on working memory, giving rise to what is known as the split-attention effect. According to a recent theoretical conceptualization (Andersen and Makransky, 2021), the original concept of Extraneous Load has been broadened to encompass sub-dimensions related to background noise and the electronic devices used to process learning material; (iii) Germane Cognitive Load is defined as the mental resources needed to encode new information into long-term memory, specifically addressing intrinsic cognitive load rather than extraneous cognitive load. Our work involved applying two different levels of Extraneous load to the task: at the low level, we used a cognitive interference paradigm alone. This paradigm includes supplementary information that demands attention as a part of a secondary load task, consequently imposing a burden on working memory. This occurs when individuals not only focus on the objectives of the primary task but also on those of the secondary load task (e.g., Janowich et al., 2015). In contrast to the previous cognitive load paradigm employed in moral decision-making involving accidental harm, which incorporates a verbal secondary task while individuals watch a video presenting a moral scenario (e.g., Buon et al., 2013; Martin et al., 2021), we used a visual secondary task. This was done because the KE material is usually presented verbally and lacks visual representation. Moreover, at a higher level, it took advantage of the combination of two different experimental manipulations: the cognitive interference paradigm and a time pressure paradigm. This required participants to complete the task within a tight deadline (e.g., Raoelison et al., 2020; Bago et al., 2021). This approach can enhance our comprehension of how various levels and types of extraneous cognitive load impact the decision-making surrounding intentionality attribution.

### 1.4 Aims and hypotheses

The study aims to investigate the impact of two different levels of extraneous cognitive load on a particular type of decision-making, which involves determining intentionality. We will compare the traditional Dual Process Model theory with the newer Dual Process Model 2.0. Specifically, we will examine how overloaded cognitive resources influence decision-making when confronted with the Knobe scenario. To accomplish this, we initially conducted a study that aimed to decrease cognitive resources by introducing a concurrent task, specifically a dot matrix task. This task places substantial demands on the participants’ executive resources (e.g., De Neys and Verschueren, 2006; Franssens and De Neys, 2009; Miyake et al., 2001). This was done to ensure a low level of Extraneous load. Indeed, previous studies have shown that concurrent tasks (e.g., Raoelison et al., 2020; Bago et al., 2021) do not always exhaust all the available cognitive resources. Therefore, a second study was conducted to test participants who were forced to respond within a challenging deadline while performing the secondary task (high level of Extraneous load). The latter procedure has been widely used (e.g., Bago and De Neys, 2017; Bago and De Neys, 2019a; Bago and De Neys, 2019b; Raoelison et al., 2020; Bago et al., 2021) to achieve complete exhaustion of the available resources and to elicit a truly intuitive response in the natural context, considering that deliberation is known to require time and resources (Kahneman, 2011; Kahneman and Frederick, 2005). According to the classic Dual Process Model, the first hypothesis (Hy1) of Study 1 suggests that by increasing the cognitive load on the decision-making process with a dual-task paradigm, the intuitive response will dominate, leading to a more pronounced asymmetry in attributing intentionality. As a result, the amount of intentionality attributed to negative side effects will increase, while it will decrease for the positive side effects. Regarding the negative scenario, the intuitive response coincides with a reasoning heuristic that leads to the misattribution of intentionality. This holds true in both the classical and 2.0 Dual Process models. Similar findings have been identified in moral dilemmas, where the cognitive load has led individuals to weigh causation more heavily than the actor’s intentions when judging the morality of an accidental action (Buon et al., 2013; Timmons and Byrne, 2019; Martin et al., 2021). On the other hand, in the positive scenario, the heuristic response aligns with the logical one because there was no intention to cause the side effect. Therefore, we hypothesized that the heuristic response leads to a decrease in ascribed intentionality. According to the 2.0 Dual Process model, this decrease could result from System 1 (logical intuition) if no difference in response time between negative and positive side effects is detected. Conversely, if participants require more time to respond to the positive side effect, then this decrease would likely result from System 2 (deliberation), as proposed by the classical Dual Process model. Indeed, as in previous experiments on moral cognition, the collected reaction times during the task indicate that longer times lead to more deliberate judgments based on System 2. In contrast, shorter response times are linked with more intuitive responses influenced by System 1 (Greene et al., 2001, 2008). In this case, we can expect a similar association, supporting the second hypothesis: shorter response times will be connected to more intuitive, intentional responses. Specifically, faster response times are anticipated for the negative side effects compared to the positive ones due to a switch from System 1 to System 2 according to the classic Dual Process Model for giving a logical response. Meanwhile, we do not anticipate any differences in times according to the Dual Model 2.0, having the positive side effect of a prompted logical response (Hy2). In Study 2, we introduce time pressure to the dual-task paradigm to magnify the cognitive load, due to the lack of consensus in the literature regarding whether this paradigm alone sufficiently depletes all available cognitive resources to generate a substantial cognitive load (e.g., Bago and De Neys, 2017; Bago and De Neys, 2019b; Raoelison et al., 2020; Bago et al., 2021). We expect that having a high Extraneous cognitive load again highlights the reliance on intuitive heuristics. This will lead to a greater inclination to attribute intentionality to negative side effects and a decreased inclination to attribute intentionality to positive side effects. Moreover, the time limit will necessitate that both positive and negative intentionality responses be driven by System 1 thinking. This is because insufficient time is available for more thorough reasoning and contemplation of the responses. In this situation, the positive side effects should be credited to System 1, along with the negative side effect, according to Dual Process Model 2.0, finding consequently no differences in response times between the negative and positive side effect in the conditions with time constraints (Hy3). Regarding response times, as a manipulation check, we expect in the condition without load, longer response time than in the condition under high extraneous load (secondary load task and time constraint) (Hy4).

## 2 Experiment 1

### 2.1 Materials and methods

#### 2.1.1 Participants

A power calculation was performed to determine the sample size using G*Power 3.1. (Faul et al., 2007). To perform ANOVA analyses considering six groups (3 cognitive load levels, high vs. low vs. control X 2 valence levels, positive vs. negative, for more details, see Procedure) and the following parameters (effect size f2 = 0.30 - medium magnitude; alpha = 0.05; power = 0.90), the required sample size was at least 192 participants. The original sample consisted of 224 participants (age *M* = 23.79, *SD* = 1.75, education *M* = 15.70, *SD* = 2.05, 42% males), who were randomly divided into the six experimental conditions (for more details, see Procedure) through the Qualtrics platform (First release: 2005, Provo, Utah, USA^1^). Twenty-eight participants were excluded because they made more than two errors in the concurrent task, which means that the task had not been sufficient to reduce their cognitive resources (see Materials and Result section for more details). The final sample consisted of 195 participants (age *M* = 23.69, *SD* = 1.69, education *M* = 15.61, *SD* = 2.16, 43.1% males). The demographic statistics for each experimental group are presented in Table 1.

**Table 1:** Demographic statistics of the samples (i.e., means and standard deviations: S.D).

Valence	Load	Participants	Males	Age mean	Age S.D.	Education Mean	Education S.D.
Negative	High	32	14	24.00	1.54	15.53	1.77
	Low	31	16	23.54	1.60	15.74	1.54
	None	33	10	23.36	1.55	15.06	2.64
Positive	High	34	13	23.52	1.77	15.82	2.18
	Low	32	16	24.03	2.25	15.65	1.87
	None	33	15	23.69	1.31	15.81	2.66

Participants were recruited from an Italian university campus and citizen associations using notices on social networks and bulletin boards. Participation required the absence of a history of major neurological or psychiatric disorders. No participants reported having been diagnosed with such disorders, so none were excluded for this reason. This was assessed through a brief medical history survey in which participants were asked to indicate whether they had been diagnosed with any relevant conditions. Data were collected from August 2021 to November 2021. All participants provided written informed consent to participate. The study protocol received approval from the local Ethics Committee (Prot. no. 130861, University of Bologna, Italy).

#### 2.1.2 Materials

##### 2.1.2.1 Scenarios

Participants judged intentionality on 16 scenarios selected from a set of 80 (Ngo et al., 2015) based on Knobe’s original scenario (Knobe, 2003). Each scenario described the action of a leading character, which resulted in a side effect. Eight scenarios involved a negative side effect (i.e., harming someone/something; e.g., The farmer’s pesticide harmed his neighbour’s crop), and eight corresponding scenarios involved a positive side effect (i.e., benefiting someone/something; e.g., The farmer’s antifungals protected his neighbour’s crop; see Supplementary material for the whole set of scenarios).

##### 2.1.2.2 Dot memorization task – dual-task paradigm (*extraneous* cognitive load)

**Figure 1:**
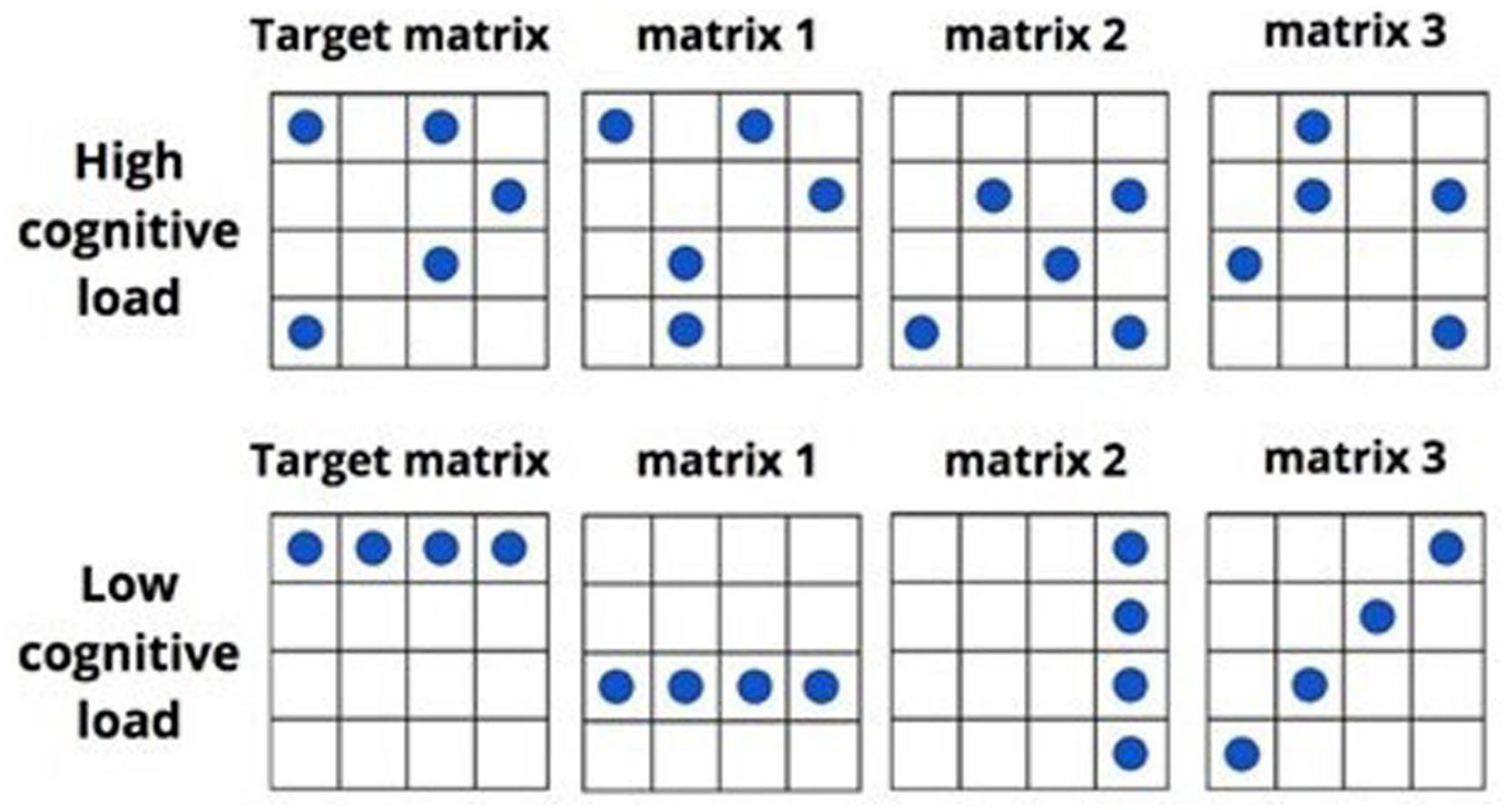
Examples of the matrices and recall options for HLC and LLC [adapted from: De Neys and Bialek (2017)].

The dual-task paradigm used in the study was based on the dot memorization task (Miyake et al., 2001; De Neys and Bialek, 2017). Participants were required to memorize dot patterns within a square grid and identify them among four alternatives. This task was selected to heighten cognitive load, as it is recognized to tax participants’ executive resources (De Neys and Verschueren, 2006; Franssens and De Neys, 2009; Miyake et al., 2001). There were two types of matrices based on the cognitive resource load: in the high load condition (HLC), the matrix had five dots arranged in a complex interspersed pattern in a 4 × 4 grid; in the low load condition (LLC), the matrix had a simple pattern of four aligned dots, which should be easier on executive resources (De Neys, 2006; De Neys and Verschueren, 2006). All response options in the HLC exhibited a pattern with 5 dots arranged in an interspersal manner. Interestingly, there was consistently a single incorrect matrix among the four options that closely resembles the correct matrix by sharing three out of the five dots (e.g., see Figure 1, matrix 1). Furthermore, the remaining two incorrect matrices only shared a single dot with the correct matrix (e.g., see Figure 1, matrices 2 and 3). In contrast, the LLC presented a distinct arrangement where all response options showcased four dots aligned in a straight line.

#### 2.1.3 Procedure

The experiment took place online using the Qualtrics platform [first release: 2005, Provo, Utah, USA (see Footnote 1)] as there were social restrictions due to the COVID-19 pandemic. Participants were invited to join the experiment through email or social networks. Once they agreed, they had a video call with the experimenter. The experimenter illustrated the research project and remains available during the experiment to address any doubts the participant had. Participants were randomly assigned to one of the six experimental conditions, each with varying cognitive loads (HLC vs. LLC vs. no cognitive load) and scenario valence (negative vs. positive). This resulted in a 3 × 2 between-subject design. All participants received clear instructions about the procedure before the experiment began.

**Figure 2:**
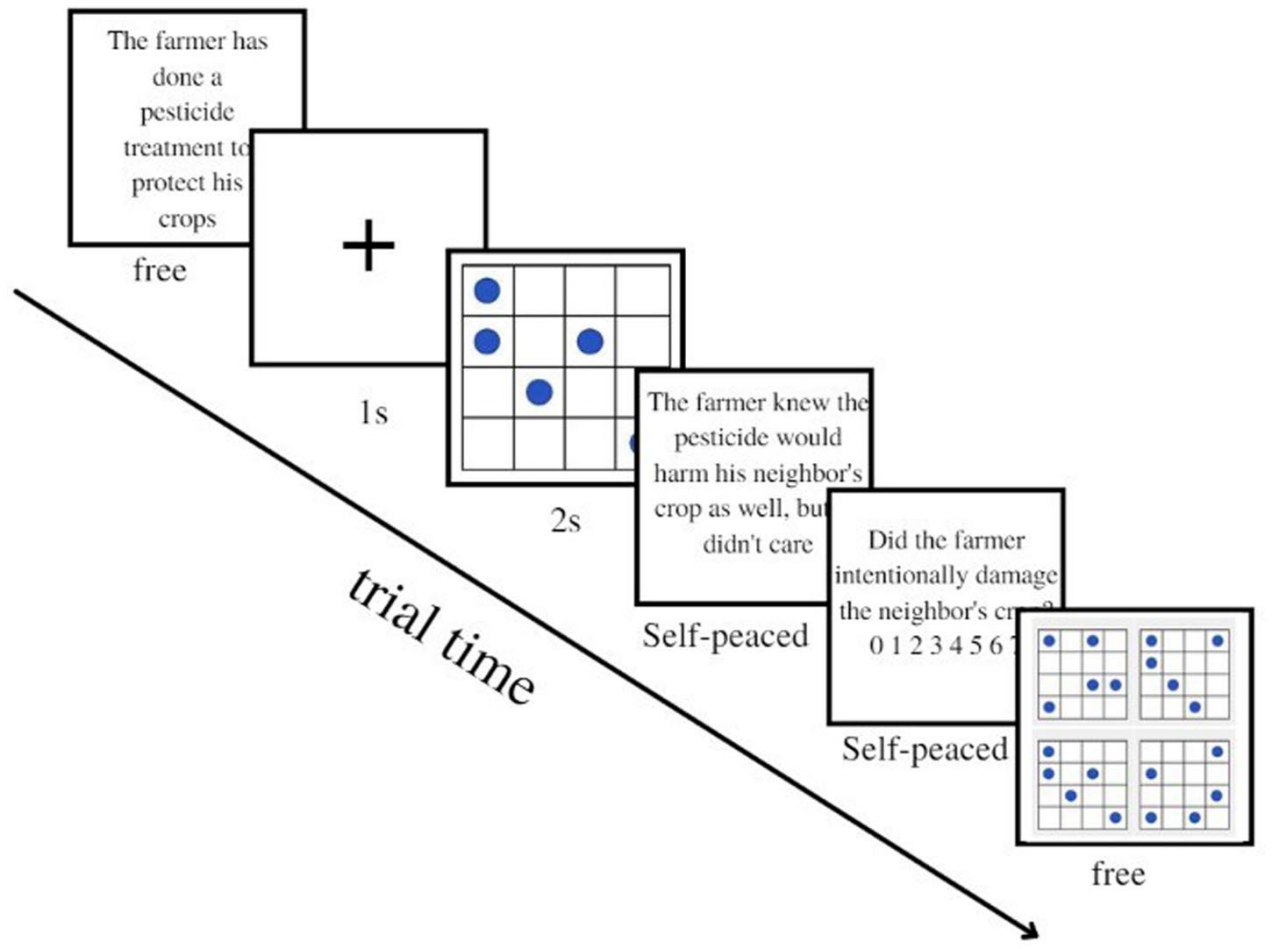
Example of experimental trial (HLC, negative scenario).

In conditions with cognitive load using the dot memorization load task, we followed the procedure outlined by De Neys and Bialek (2017). Initially, participants were presented with two simplified dot memorization practice trials to familiarize them with the procedure. In the practice trials, participants were presented with a simple arithmetic problem instead of a Knobe scenario, alternating with an easy dot matrix. Then, participants started the experiment, and each participant was administered eight scenarios and eight matrices. The procedure was as follows (De Neys and Bialek, 2017): participants were shown the first part of a scenario, describing the context in which the action takes place. On the next page, the target matrix to be memorized appeared and remained visible for 1 s in the LLC and for 2 s in the HLC. The matrix was always preceded by a fixation cross lasting 1 s. Once the matrix disappeared, participants were given the second part of the scenario, containing the critical information about the action performed and its side effects. After participants read the second part of the scenario, they were asked to ascribe intentionality to the side effect described using a Likert scale from 0 (not at all) to 7 (completely). On the following page, participants had to recognize the memorized target matrix from among four alternatives. Once the matrix was selected, participants moved to the next scenario (see Figure 2). This procedure was repeated for all eight scenarios in each cognitive load condition. Scenarios were shown in two parts to reduce the amount of information to recall under cognitive load, as suggested by De Neys and Bialek (2017). In the control condition, the eight scenarios were presented following the same procedure through the Qualtrics software, except for the absence of the dot matrices, with scenarios shown alternately with blank screens. During intentionality ascription, response time was collected to detect task performance with and without cognitive load. The order of the scenarios was randomized in every experimental condition.

### 2.2 Results

#### 2.2.1 Dual-task paradigm (*extraneous* cognitive load)

We followed the procedure outlined by De Neys and Bialek (2017). For this task, we excluded participants who made more than two errors in recalling the memorized matrix (i.e., less than 75% accuracy). This was done to ensure that the task placed an effective burden on executive resources. Among participants who performed the task under cognitive load, 28 participants were excluded because they exceeded the maximum number of errors allowed (see Table 1 for the final number of participants in each condition and specific demographics). Age, education, and gender had no significant impact on intentionality attribution or log-transformed response times. Six mixed-effects models were employed: three for intentionality and three for response times. Each model included valence (2 levels, positive, negative) and cognitive load (3 levels, high, low, no-load) as fixed effects, with participants as a random effect. Age and education were included as covariates in two models, while gender was included as a fixed factor in the third model for each dependent variable. No significant main effects or interactions involving these covariates or gender were observed. For additional details, refer to the Supplementary materials.

#### 2.2.2 Intentionality scores

**Figure 3:**
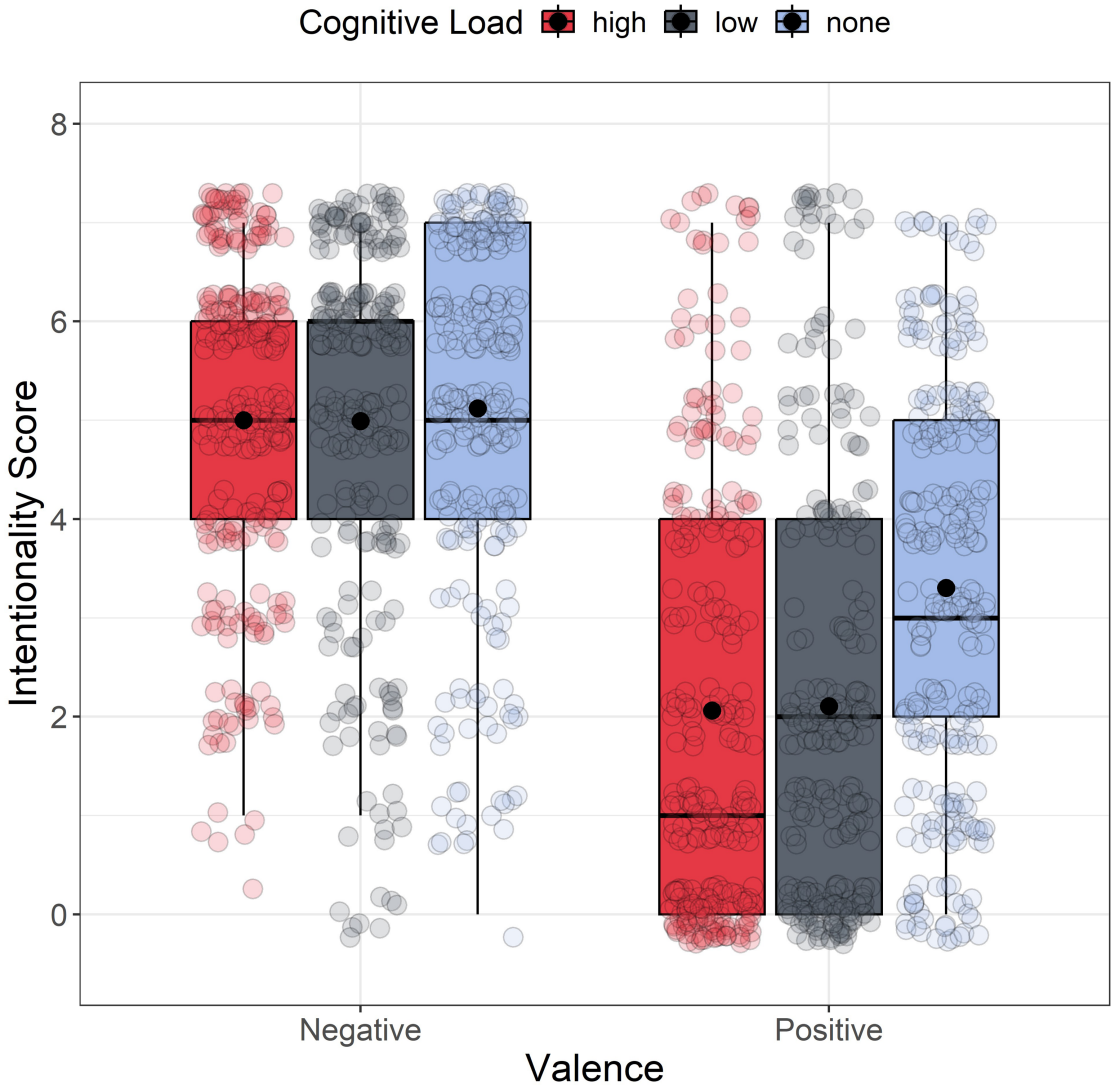
Boxplot of intentionality scores as a function of the valence. For each boxplot, the horizontal black line indicates the median, the boxes in the boxplots extend from the lower quartile to the upper quartile (i.e., the 25th and 75th percentiles). The dot within each boxplot represents the mean intentionality value for each level of the cognitive load.

Analyses were performed using R (version 4.4.1, 2024) and RStudio (2023.06.0, build 421). Figure 3 shows the boxplot of intentionality scores for each group. We used ‘lme4’ and ‘ARTool’ packages to perform a non-parametric factorial analysis on intentionality scores. Given that the assumption of normality of residuals was not met for all groups, we decided to perform a non-parametric factorial analysis using the Aligned Rank Transform (ART) method from the R package ‘ARTool’ (Elkin et al., 2021; Wobbrock et al., 2011). We applied ART with a linear mixed model considering valence (negative vs. positive) and cognitive load (high, low, and none) as fixed between-subjects factors. As a random effect, we included the by-subject and by-scenario random intercepts. The full model was:


(1) $$int\_\mathrm{score}\sim \mathrm{Valence}*\mathrm{Load}+\left(1|S\right)+\left(1\mathrm{|Scenario}\right)$$

Please refer to the Supplementary material for a more detailed discussion on the verification of assumptions.

ART is a non-parametric technique designed to analyze data robustly, particularly when assumptions such as normality of residuals and equality of variances are violated. In ART, the data in each group are ranked from lowest to highest, ensuring that each data point receives a rank based on its value relative to others in the group. These ranks are then aligned across different groups by matching ranks of the same value, which helps to mitigate issues arising from varying scales or variances between groups. After alignment, the ranks are transformed back to the original scale of the data, allowing for the application of traditional statistical methods such as ANOVA or linear mixed models. This transformation ensures that the data can be analyzed accurately while accounting for the alignment of ranks across groups. By using the ART method, researchers can conduct robust and reliable analyses, even in situations where the data deviates from standard assumptions of normality and equal variances. *Post hoc* analysis was conducted using the ‘art.con’ function (Elkin et al., 2021; Kay et al., 2021) and effect sizes were estimated following the procedure described by Elkin et al. (2021).

The analyses revealed a significant main effect of valence *F*(1, 189) = 116.853, *p* < 0.001, *ηp^2^* = 0.382, indicating that participants had significantly higher intentionality scores in the negative valence condition (*M* = 5.039, *SD* = 1.771) than in the positive valence condition (*M* = 2.490, *SD* = 1.206). Additionally, a significant main effect for cognitive load was observed *F*(2, 189) = 5.360, *p* = 0.005, *ηp^2^* = 0.054, along with a significant interaction between valence and cognitive load: *F*(2, 189) = 3.373, *p* = 0.036, *ηp^2^* = 0.034. Post hoc comparisons adjusted using the FDR method (Benjamini and Hochberg, 1995), revealed significant differences between the high-load (HLC) and low-load (LLC) conditions when compared to the no-load condition (*p_adj_* = 0.009 for both comparisons). Conversely, no significant difference was observed between the two load conditions (*padj* = 0.923). Participants attributed lower intentionality to side effects in both HLC (*M* = 3.487, *SD* = 2.431) and LLC (*M* = 3.526, SD = 2.491) relative to the no-load condition (*M* = 4.212, *SD* = 2.126). Furthermore, FDR-corrected *post hoc* comparisons for 15 tests revealed significant differences within the positive condition. In the positive condition, scenarios presented under HLC (*M* = 2.062, *SD* = 2.183) and LLC (*M* = 2.105, *SD* = 2.177) were perceived as less intentional compared to the scenario presented in the no-load condition (*M* = 3.303, *SD* = 2.036) (*p_adj_* = 0.007 and *p_adj_* = 0.004, respectively). No significant results have been found within the negative condition (*p_adj_* = 0.841 and *p_adj_* = 0.944, respectively).

#### 2.2.3 Response times

**Figure 4:**
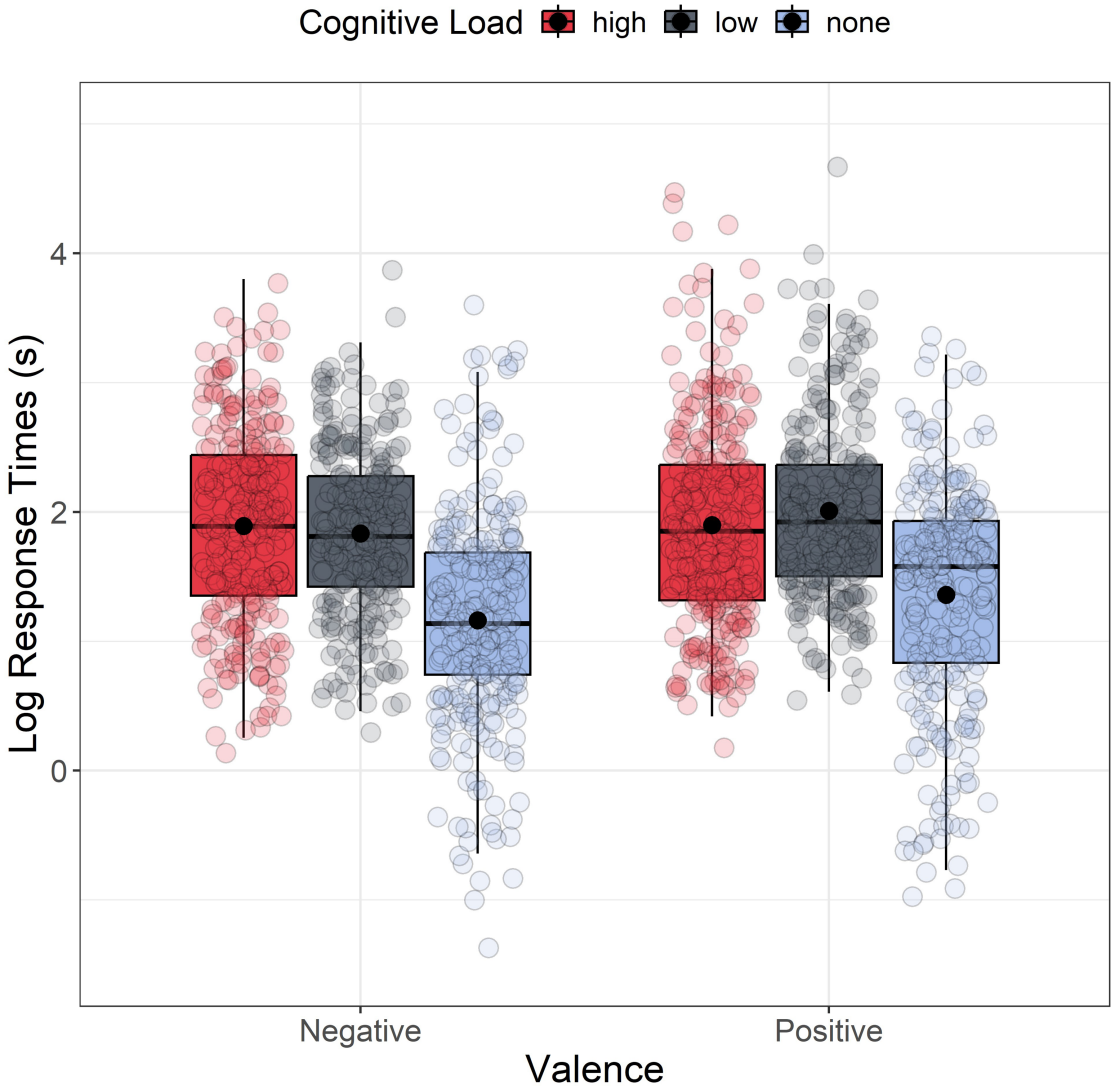
Boxplot of response times as a function of the valence. For each boxplot, the horizontal black line indicates the median. The dot within each boxplot represents the mean response time for each level of the cognitive load.

Figure 4 shows the response times results. For each scenario, response time represents the time elapsed between the presentation of the question and the participants’ selection of their answers. Descriptive analyses (i.e., normality of residuals, skewness, kurtosis, the presence of outliers, and violated homogeneity of variances) indicated a significant departure from normality for all groups. Therefore, response times were log-transformed before analysis, following the approach of previous studies (Bago et al., 2021; De Neys and Bialek, 2017). However, even after log transformation, the assumptions of normality of residuals and homogeneity of variances were not met. Given this, we again used a non-parametric factorial analysis using the Aligned Rank Transform (ART) method with a linear mixed model including valence (negative vs. positive) and cognitive load (high, low, and none) as fixed between-subjects factors and random intercepts across subjects and scenarios (see Equation 1). The analysis showed a marginally significant main effect of Valence, *F*(1, 189) = 3.649, *p* = 0.057, *ηp^2^* = 0.019, suggesting that participants took less time answering to negative side effects (*M* = 6.952, *SD* = 5.964; log(*M*) = 1.621, log(*SD*) = 0.825) than to the positive ones (*M* = 8.249, *SD* = 9.089; log(*M*) = 1.753, log(*SD*) = 0.836). The analysis also revealed a significant main effect of the Cognitive Load: *F*(2, 189) = 37.279, *p* < 0.001, *ηp^2^* = 0.283. FDR-corrected post hoc comparisons indicated significant differences between the two load conditions (HLC and LLC) and the no-load condition (both with *p_adj_* < 0.001) but no significant difference between the two cognitive load conditions (*p_adj_* = 0.923). Participants took significantly more time to answer in both HLC (*M* = 9.057, SD = 9.072; log(*M*) = 1.895, log(*SD*) = 0.757) and LLC (*M* = 8.781, *SD* = 8.001; log(*M*) = 1.922, log(*SD*) = 0.677) compared to the no-load conditions (*M* = 5.046, *SD* = 4.858; log(*M*) = 1.261, log(*SD*) = 0.874) (Figure 4).

### 2.3 Discussion

This study’s aim was to explore the effect of the extraneous cognitive load using a dual-task paradigm on the Knobe Effect (KE; Knobe, 2003). Specifically, our hypothesis entailed that placing an additional burden on cognitive resources, through the imposition of a cognitive load when ascribing intentionality, would intensify the asymmetry in intentionality attribution between positive and negative side effects, raising intentionality judgments for negative outcomes and lowering them for positive ones (Hy1). Our results support our hypothesis, but only for the positive side effect. We found that participants ascribed less intentionality to the positive side effect under both high and low cognitive load. Our analyses did not reveal any significant difference within the negative condition.

Looking at the results in more detail, we observed that in the cognitive load conditions, response time was significantly longer than in the control condition. Unlike previous studies on moral dilemmas (e.g., Conway and Gawronski, 2013; Trémolière and Bonnefon, 2014), the presence of a dual task alone was not enough to elicit a high cognitive load. In this case, dealing with the secondary load task caused the participants to take more time to think, weakening our manipulation. Our analysis also showed a marginally significant main effect of valence on response times, with participants taking less time to respond to the negative side effects than the positive ones, confirming our second hypothesis (Hy2).

It is well-known in literature how, in the KE, negative side effects elicit a strong emotional response (Ngo et al., 2015; Zucchelli et al., 2019). Faster response times for negative side effects could be the result of a System 1-like response, which we assume could be one of the main reasons why the manipulation only found significant results for the positive side effects. If we already have a System 1 fast response for negative side effects, a stronger manipulation would be needed to observe a difference. Instead, the manipulation lowered the intentionality attribution scores for the positive side effects, even though participants took slightly longer to respond. The prolonged duration and thorough contemplation of the response may suggest that the rational reaction to a positive situation, which has a lower emotional impact, is linked to System 2. This system is easily affected by cognitive load as it relies on cognitive resources. Including a time restriction in the second experiment should address and clarify any existing uncertainties.

## 3 Experiment 2

### 3.1 Aim and hypotheses

The purpose of the second experiment is to investigate whether a higher extraneous cognitive load, particularly time pressure associated with the dual-task paradigm, would effectively exhaust all the cognitive resources necessary for deliberation, thereby affecting intentionality ascription for both positive and negative side effects. Our hypothesis suggests that there will be a significant difference in the attribution of intentionality between positive and negative side effects (Hy1). Specifically, we predict that participants will be more likely to attribute intentionality to negative side effects and less likely to attribute it to positive side effects. Additionally, we believe that manipulating the time limit will help us understand if participants’ responses to both positive and negative effects are driven by their intuitive, automatic thinking (System 1). The limited time will prevent participants from further reasoning about their responses. We plan to test this by comparing the response times of positive and negative side effects when the time constraint is applied (Hy3). Furthermore, as a manipulation check, we expected that individuals in the condition without cognitive load (and thus without time constraints) would take longer to attribute intentionality to both positive and negative side effects compared to HLL and LLC (Hy4).

### 3.2 Materials and methods

#### 3.2.1 Participants

The second experiment had the same design as the first one, requiring a minimum of 192 participants (G*Power 3.1. calculation; Faul et al., 2007). The original sample consisted of 276 participants (age *M* = 23.58, *SD* = 4.16; education *M* = 14.80, *SD* = 2.65; 35.5% males) who were randomly assigned to six experimental conditions (3 cognitive load levels: HLC vs. LLC vs. control x 2 valence levels: positive vs. negative) using the Qualtrics platform (First release: 2005, Provo, Utah, USA). Thirty participants were excluded because they made more than two errors in the concurrent load task and thirteen participants were excluded because they exceeded the imposed time constraint (see Procedure and Results section for more details). Participants were excluded, in both cases, because their cognitive resources had not been reduced effectively, as required by the cognitive load condition. The final sample consisted of 233 participants (age *M* = 23.65, *SD* = 4.44; education *M* = 14.84, SD = 2.45; 36.1% males). The demographic statistics for each experimental group are presented in Table 2.

**Table 2:** Demographic statistics (means and standard deviations S.D).

Valence	Load	Participants	Males	Age mean	Age S.D.	Education mean	Education S.D.
Negative	High	41	12	23.09	3.17	14.78	1.86
	Low	36	14	24.40	5.78	14.16	3.18
	None	45	18	23.98	3.12	15.38	2.42
Positive	High	36	15	22.89	5.01	14.72	2.26
	Low	40	14	23.12	2.95	14.90	3.34
	None	35	11	24.48	5.71	14.94	2.56

Participants were recruited from an Italian university campus and through citizen associations by posting notices on social networks and bulletin boards. Participation required the absence of a history of major neurological or psychiatric disorders. No participants declared any diagnosis in such conditions, so none were excluded for this reason. This was again confirmed through a brief medical history survey. Data collection took place from November 2022 to March 2023, during which every participant provided written informed consent to take part in the experiment.

#### 3.2.2 Procedure

Since social restriction due to COVID-19 pandemic were over, Study 2 was conducted in presence. This transition provided an opportunity to enhance our experimental control, particularly for the stringent time–pressure manipulation of second study (6 s), more reliably implemented in a laboratory setting. Anyway, we ensured methodological consistency by using the same experimental platform [Qualtrics - First release: 2005, Provo, Utah, USA (see Footnote 1)] and comparable task designs across both studies.

**Figure 5:**
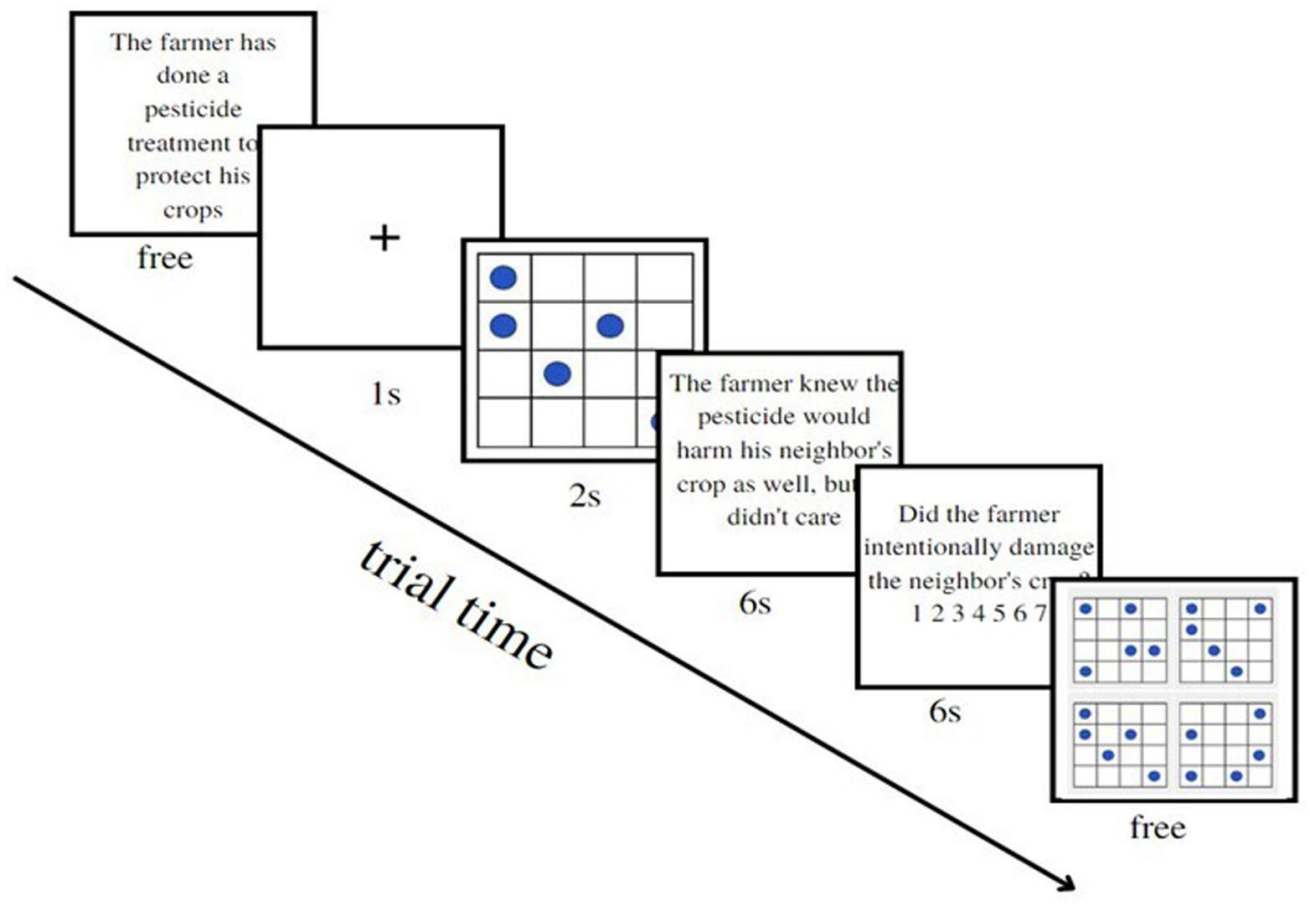
Example of experimental trial with time limit procedure of 6 s (HLC, negative scenario).

A pre-test was performed initially to determine the appropriate time pressure for the experimental procedure. During this pre-test, participants were instructed to read and answer the second part of the scenario as quickly as possible. This section contained critical information about the action performed and its consequences, as well as the intentionality question. The aim was to determine the average time required to perform the task. Participants needed an average of 6 s to read the second part of the scenario and the intentionality question; which aligns with findings from other studies on moral cognition (e.g., Hashimoto et al., 2022; Tinghög et al., 2016). The pre-test details can be found in the Supplementary materials. Participants were then invited to take part in the experiment via email or social media. They were welcomed into the university laboratory, where the experimenter explained the research project and was available throughout the experiment. Participants were randomly assigned to one of six experimental conditions. The procedure was similar to Study 1, with the addition of a time pressure paradigm. During the intentionality judgment task, participants had to respond within 6 s, unlike the previous experiment, where they had unlimited time. As in Experiment 1, participants were first presented with two simplified dot memorization practice trials to familiarize themselves with the procedure and added time constraint. Before the experiment, participants were instructed about the 6-s time limit, and informed that a red notice stating “answer this question” would appear when approaching the time limit (at 5 s). All responses were collected, and those exceeding the 6-s time limit were discarded (see participants section). Participants again provided intentionality judgments on a Likert scale from 0 (not at all intentional) to 7 (totally intentional). Afterward, they had to identify the memorized target matrix from four alternatives described in the Materials section of Study 1. Once the matrix was selected, participants moved on to the next scenario. This procedure was repeated for all sixteen scenarios and for each participant in every cognitive load condition (see Figure 5 for a procedure example). In the control condition, the procedure was the same except for the absence of the high level of *extraneous* cognitive load (dual task paradigm + time pressure). In this condition, scenarios alternated with blank screens, and participants had unlimited time to ascribe intentionality. To assess the impact of the time–pressure manipulation, we recorded participants’ response times in the load and control conditions while they made intentionality judgments. The order of scenarios in each experimental condition was randomized to avoid any potential order effect.

### 3.3 Results

#### 3.3.1 Dual-task paradigm and time limit (extraneous cognitive load)

As in Study 1, we followed the procedure outlined by De Neys and Bialek (2017) by excluding participants who made more than two errors while recalling the memorized matrix. This step was crucial to ensure that the cognitive load task effectively burdened executive resources, resulting in an accuracy rate of more than 75%. Out of the participants who completed the task under cognitive load, 30 participants were excluded because they exceeded the maximum allowable number of errors. Additionally, 13 participants were unable to provide intentionality judgments within the 6-s time limit. The final sample is described in Table 2 (i.e., the number of participants in each condition and demographic statistics). As in study 1, age, education, and gender had no significant impact on intentionality attribution or log-transformed response times. Six mixed-effects models were employed: three for intentionality and three for response times. Each model included valence (2 levels: positive, negative) and cognitive load (3 levels: high, low, no-load) as fixed effects, with participants as a random effect. Age and education were included as covariates in two models, while gender was included as a fixed factor in the third model for each dependent variable. No significant main effects or interactions involving these covariates or gender were observed. For additional details, refer to the Supplementary materials.

#### 3.3.2 Intentionality scores

**Figure 6:**
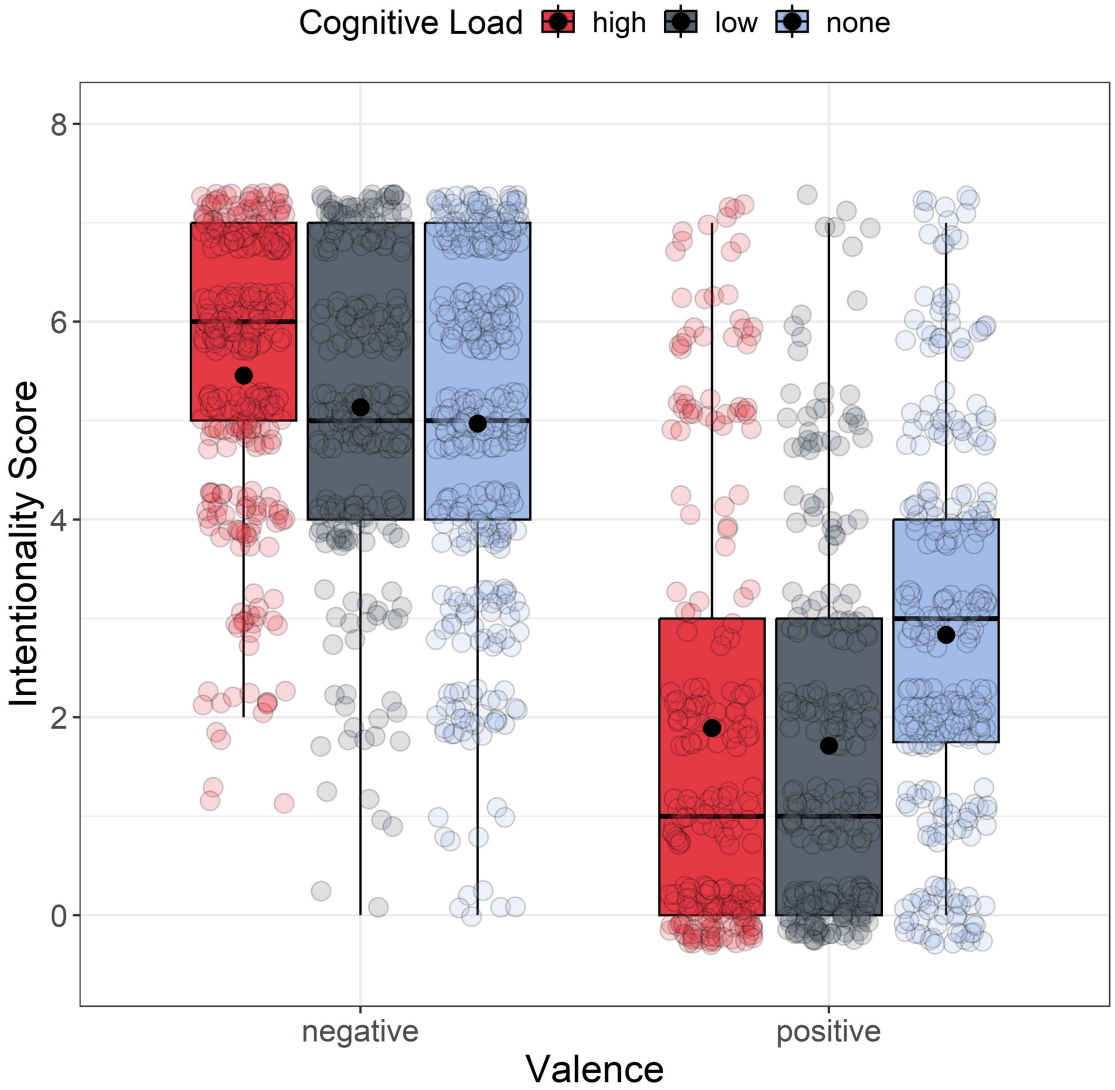
Boxplot of intentionality scores as a function of the valence. For each boxplot, the horizontal black line indicates the median. The dot within each boxplot represents the mean intentionality value for each level of the cognitive load.

We followed the same procedure of Study 1. Figure 6 shows the boxplot of intentionality scores for each group. The QQ plot and skewness/kurtosis values indicated a non-normal distribution of residuals for all of our groups. The significant Levene’s test (*F*(5, 1,663) = 12.521, *p* < 0.001) confirmed variance heterogeneity, with no outliers detected. Therefore, we performed again a non-parametric factorial analysis, applying the Aligned Rank Transform (ART) method to a mixed linear model, with valence (negative vs. positive) and cognitive load (high, low, and none) as between-subjects factors and by subject and scenario random intercept (see Eq. 1). The analysis showed a significant main effect of valence *F*(1, 227) = 245.034, *p* < 0.001, *ηp^2^* = 0.519, suggesting that participants had significantly higher intentionality scores in the negative valence (*M* = 5.178, *SD* = 1.638) than in the positive valence side effect (*M* = 2.179, *SD* = 2.081), and a main effect for load *F*(2, 227) = 4.433, *p* = 0.013, *ηp^2^* = 0.038. FDR-corrected *post hoc* comparisons showed how participants generally assigned lower levels of intentionality attribution to side effects in LLC (*M* = 3.400, *SD* = 2.445) compared to the no-load condition (*M* = 4.038, *SD* = 2.150). However, we also found a significant interaction between valence and load *F*(2, 227) = 6.829, *p* < 0.001, *ηp^2^* = 0.057. FDR-corrected post hoc comparisons for 15 tests revealed significant differences within the positive condition. In the positive condition, scenarios presented under high cognitive load (*M* = 1.893, SD = 2.204) and low cognitive load (*M* = 1.714, *SD* = 1.895) were perceived as less intentional compared to the scenario presented in the no-load condition (*M* = 2.836, *SD* = 1.968) (with *p_adj_* = 0.022 and *p_adj_* = 0.001 respectively). Also, this time, we did not find significant difference within the negative condition, having that even though scenarios presented under HCL (*M* = 5.456, *SD* = 1.440) and LCL(*M* = 5.134, *SD* = 1.582) were considered as more intentional than the ones without cognitive load (*M* = 4.972, *SD* = 1.794), this difference have not reached statistical significance (*p_adj_* = 0.109 and *p_adj_ = 0*.712 respectively).

#### 3.3.3 Response times

**Figure 7:**
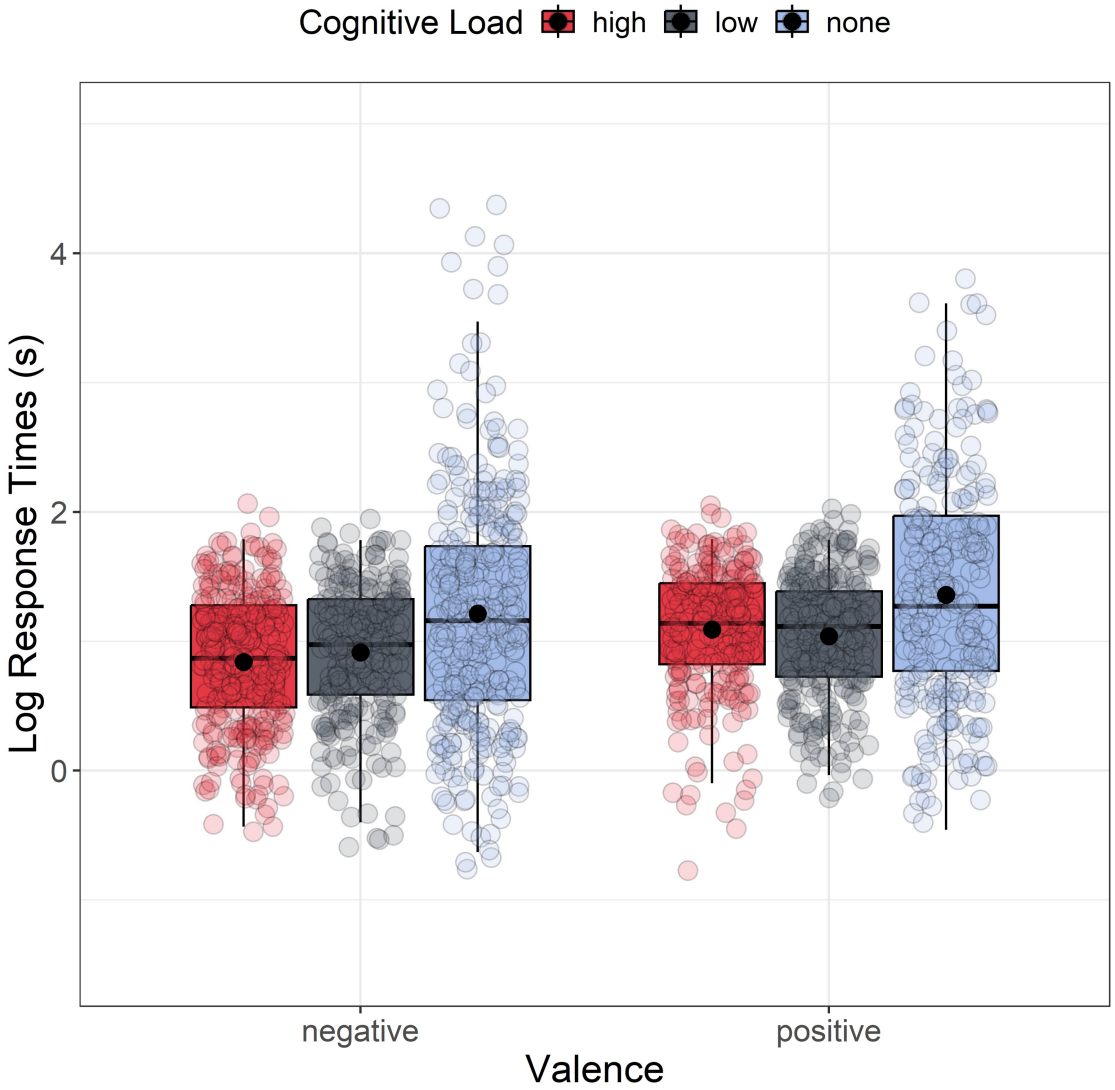
Boxplot of response times as a function of the valence. For each boxplot, the horizontal black line indicates the median. The dot within each boxplot represents the mean response latency value for each level of the cognitive load.

Figure 7 displays the response times results. The QQ plot and Shapiro–Wilk revealed a non-normal distribution of residuals across all six groups, with two groups without time limits exhibiting a significant deviation from normality. Levene’s test confirmed heterogeneity of variances (*F*(5, 1,663) = 42.9, *p* < 0.001), and multiple outliers were identified via the boxplot method. Response times were log-transformed before analysis, as in the previous experiment. Once again, we employed a non-parametric factorial analysis applying the Aligned Rank Transform (ART) method to a mixed linear model, with Valence (negative vs. positive) and Cognitive Load (high, low, and none) as between-subjects factors and by subject and scenario random intercept (see Eq. 1). The analysis revealed a significant main effect of Valence *F*(1, 228) = 13.409, *p* < 0.001, *ηp^2^* = 0.056, suggesting that participants took significantly less time to respond to negative side effects (*M* = 3.866, *SD* = 6.291; log(*M*) = 1.001, log(*SD*) = 0.711) than to the positive ones (*M* = 4.112, *SD* = 4.076; log(*M*) = 1.172, log(*SD*) = 0.644). The analysis also reported a significant main effect of the load *F*(2, 227) = 10.578, *p* < 0.001, *ηp^2^* = 0.085. FDR-corrected post hoc comparisons indicated significant differences between the two load conditions (HLC and LLC) and the no-load condition (*p_adj_* < 0.001 for each comparison) but no significant difference between the two cognitive load conditions (*p_adj_* = 0.633). Participants took significantly more time to respond in both HLC (*M* = 2.899, *SD* = 1.277; log(*M*) = 0.950, log(*SD*) = 0.506) and LLC (*M* = 2.939, *SD* = 1.253; log(*M*) = 0.977, log(*SD*) = 0.470) compared to the no-load conditions (*M* = 5.685, *SD* = 8.278; log(*M*) = 1.278, log(*SD*) = 0.884).

To test the Hy3 hypothesis, we specifically analyzed the differences in response times between the two groups with cognitive load and time limit. We performed another non-parametric factorial ANOVA using the Aligned Rank Transform (ART) method, with valence (positive vs. negative) and cognitive load (high and low) as between-subjects factors. The analysis revealed a main effect of valence *F*(1, 148) = 21.784, *p* < 0.001, *ηp^2^* = 0.128, indicating that participants took significantly less time to respond to negative side effects (*M* = 2.695, *SD* = 1.227; log(*M*) = 0.873, log(*SD*) = 0.510) than to positive side effects (*M* = 3.167, *SD* = 1.245; log(*M*) = 1.064, log(*SD*) = 0.443), even in the presence of a time constraint. We no longer observed a significant main effect of load *F*(1, 148) = 0.333, *p* = 0.564, *ηp^2^* = 0.002, or interaction between valence and load *F*(1, 148) = 2.846, *p* = 0.094, *ηp^2^* = 0.019.

### 3.4 Discussion

This second study leveraged a ‘high’ level of *extraneous* cognitive load, achieved through the combination of a dual task and a time pressure paradigm, to deplete the cognitive resources allocated to the task. The depletion of cognitive resources was expected to lead to more intuitive responses, thereby increasing the asymmetry of intentionality attribution between negative and positive side effects.

The results of this second study partially supported our hypothesis, showing significant differences in how intentionality is attributed only in positive conditions. Under a heavier e*xtraneous* cognitive load, intentionality attribution for positive side effects decreased significantly in both the HLC and LLC conditions compared to the control condition. Contrary to expectations, no differences were observed in the attribution of intentionality to negative side effects, even under high cognitive load. This may be due to a ceiling effect in the no-load condition, where judgments were already high enough to cluster near the maximum possible intentionality score, with little variance (American Psychological Association (APA), 2018). As in Study 1, participants were faster at making negative judgments, indicating the strong influence of the emotional component on negative judgments (e.g., Zucchelli et al., 2019). This suggests that the intuitive response in the positive scenario involves more deliberation (System 2) compared to negative side effects: if System 1 was involved — as it surely is for negative responses — no difference in response times would have been observed for the positive scenario. Indeed, the estimated time for an intuitive response involving System 1 is generally very rapid, ranging from a few milliseconds to a couple of seconds (e.g., Kahneman, 2011). This type of thinking is automatic and immediate, designed to provide quick responses without requiring conscious or deliberate processing. Since response times for positive scenarios were longer, it can be assumed that six seconds is enough to transition from System 1 to System 2 for the positive scenario, suggesting that the two responses rely on different mechanisms. Lastly, response times collected during the determination of intentionality were lower in the load condition compared to the control condition, confirming the effectiveness of the experimental time manipulation (Hy4).

## 4 General discussion

**Figure 8:**
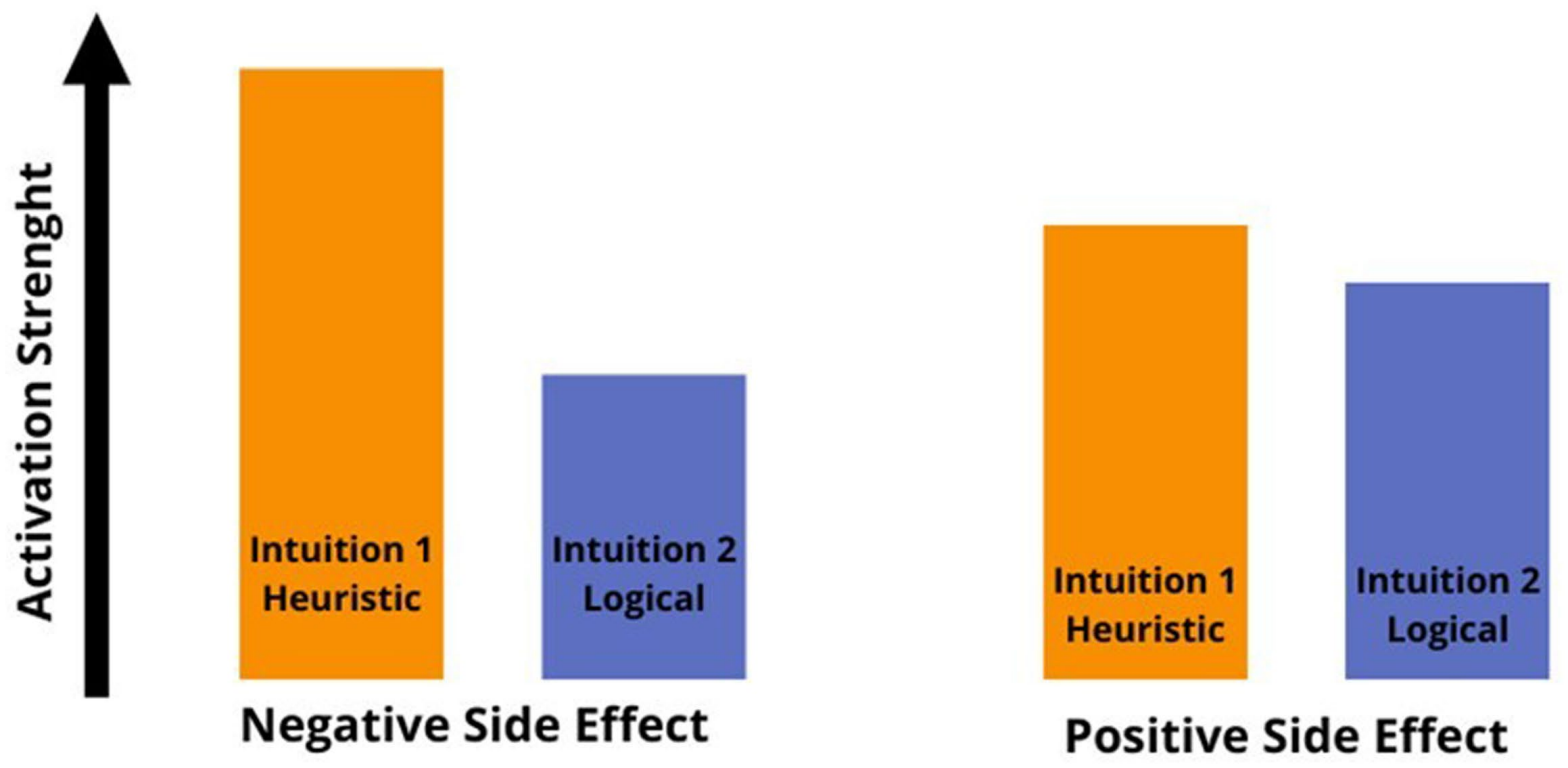
Interpretation of the Knobe Effect scenario adapted from the Dual Process model 2.0 (De Neys and Pennycook, 2019).

Problem-solving decisions can be made instantly and effortlessly or may require time and effort. The two modes of cognitive processing, known as intuitive and deliberate processing, are categorized as System 1 and System 2 in the classic dual process framework (Evans, 2008; Kahneman, 2011). De Neys and Pennycook (2019) proposed a revised version of the model, the Dual Process model 2.0, acknowledging that intuitive decision-making (System 1) can sometimes lead to heuristics use and errors, but can also produce correct and logical responses without relying on the slower, more deliberate processes of System 2, as expected in the classical model. In this study, we aimed to investigate how different levels of *extraneous* cognitive load affect decision-making about intentionality. We compared the classical Dual Process Model (Evans, 2008; Kahneman, 2011) with the Dual Process Model 2.0 (De Neys and Pennycook, 2019). Here, we focused on examining how an *extraneous* cognitive load affects individuals’ intentionality judgments when they encounter the Knobe scenarios. These scenarios propose that people attribute intentionality more to negative outcomes than to positive ones, even if both were just side effects. In Study 1, we employed a dual-task paradigm to create a cognitive load, reducing available cognitive resources. Our first hypothesis (Hy1) was only partially supported. We expected intentional attributions for negative scenarios to increase and decrease for positive ones due to cognitive load. However, we only observed this effect for positive side effects. In both the positive and negative side effect conditions of the first experiment, the response times for making intentional attributions were longer than those without cognitive load. This suggests that when people engage in the evaluation of a scenario simultaneously with a secondary load task, they tend to focus more on the decision-making task, resulting in longer response times, with a decrease in perceiving intentionality in positive situations. Additionally, positive scenarios led to longer response times compared to negative ones. Individuals in positive scenarios tend to engage in more extended reasoning and rethinking processes, while those in negative situations tend to respond more swiftly. This suggests that the response to positive side effects may not be solely due to System 1 but may also involve System 2 processes. As a result, even minor manipulations can interfere with reasoning processes as they occur. Although Study 1’s results were only partially conclusive, it showed that participants adopted a strategy to preserve their cognitive resources. Specifically, they took longer to respond, which lessened the effect of cognitive load and involved System 2 processes. Study 2 effectively reduced cognitive resources by using a dual-task paradigm under time pressure, leading to high *extraneous* cognitive load (Bago and De Neys, 2017; Bago and De Neys, 2019a; Bago and De Neys, 2019b; Raoelison et al., 2020; Bago et al., 2021). When cognitive resources are depleted due to high *extraneous* cognitive load, intuitive or emotional responses are expected to become dominant, resulting in an increased System 1 heuristic response for both negative and positive side effects. However, as in Study 1, introducing a high level of *extraneous* cognitive load intensified the ascriptions provided only for positive outcomes, in which participants denied intentions even more. In our two experiments, we found that cognitive load had different effects on positive and negative scenarios. Both studies showed that participants responded faster to negative side effects compared to positive ones. This suggests that a strong load manipulation may be necessary to influence an automatic System 1 response, as seen in negative side effects. Differently, positive side effects did not show the same quick and automatic response, as the negative side effects. When we analyzed our results in the light of the Dual Process model 2.0, we noticed differences in how strongly the heuristic response was activated in these two scenarios. In scenarios with negative side effects (as reported in Figure 8), the emotional response takes precedence over logical reasoning. Individuals swiftly and effortlessly perceive negative side effects as intentional. As a result, we have observed a diminished impact of cognitive load on negative side effects, which are inherently emotionally abrupt and heuristic in nature. However, the situation has changed for the better in terms of positive side effects. As shown in Figure 8, we observed similar activation strength between heuristic and logically intuitive responses. In the positive scenario, the logical intuition of perceiving the side effect as “not so intentional” is almost as strong as the more heuristic intuition of seeing the side effect as “not intentional at all.” According to De Neys and Pennycook (2019), in Dual Process model 2.0, people are more likely to deliberate when the activation strengths of possible different intuitions are more similar. Indeed, we have observed longer response times for positive side effects. When experiencing cognitive load, our heuristic intuition is heightened, making the distinction between it and logical intuition (as shown in Figure 8), even more striking. This promotes the intuitive heuristic to prevail more easily, reducing the likelihood of a System 2 intervention because of the thinking and rethinking processes generated by the similarity in activation strength of the two intuitions. Our results showed that participants were slower in responding to the positive scenario even when the time constraints were applied. This implies that the structure of the positive scenario, which involves two similar competing intuitions, most likely encourages a response that is similar to System 2 thinking. However, our manipulations interfered with this response.

**Figure 9:**
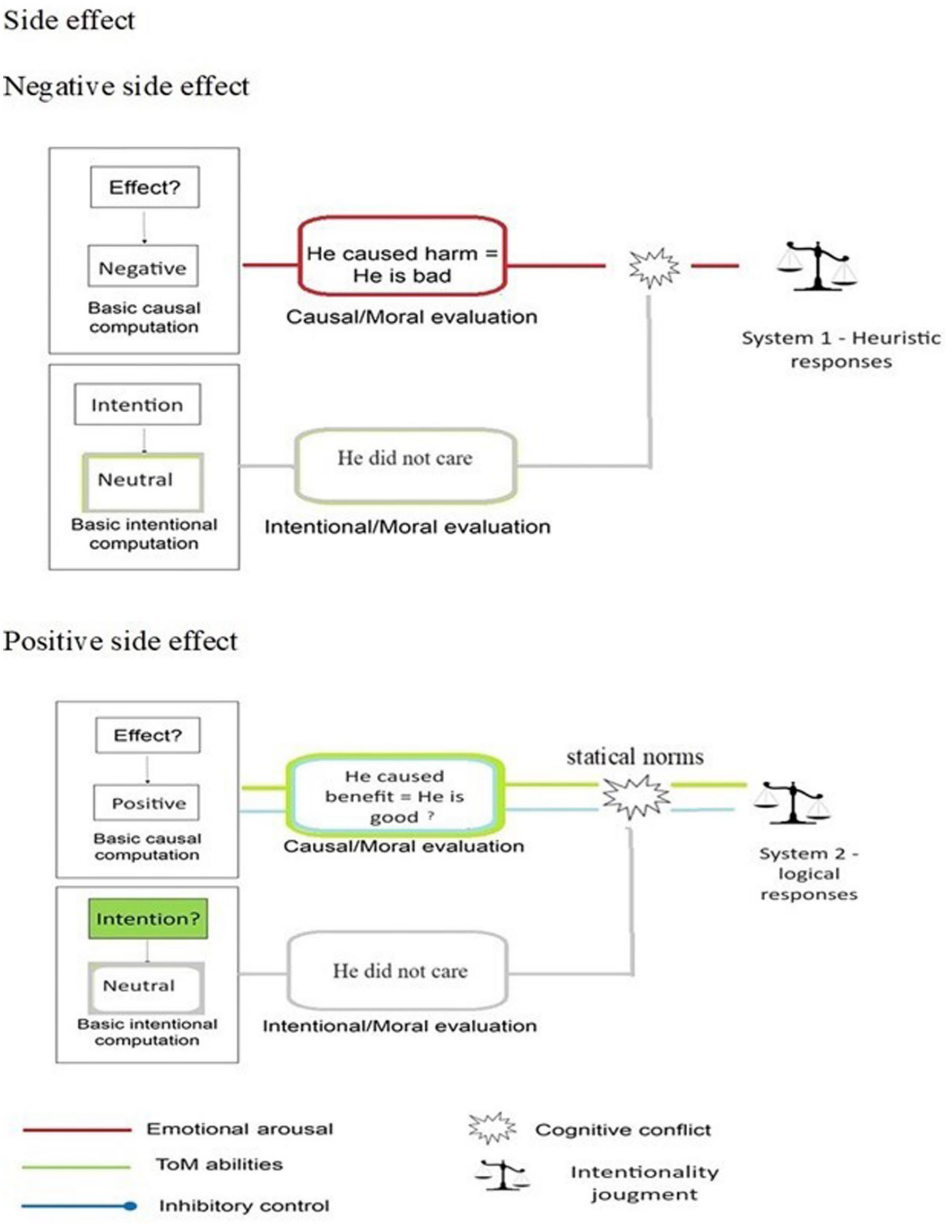
Adaptation of the ETIC (Buon et al., 2016) to the Knobe scenario considering both Dual Process models under high extraneous cognitive load.

A possible integrated explanation for this result may lie in how individuals process and combine information regarding the agent’s causal responsibility and his intention to cause harm when making moral judgments about harmful actions. This is explained by the aforementioned ETIC model (Buon et al., 2016), which proposes that the foundation of morality lies in an emotional response triggered by the recognition of a harmful action, together with the ability to understand the perspective of others (Theory of mind) and higher evaluative systems for regulating or the suppressing the emotional response (inhibitory control). According to Cushman’s (2008) proposal, two distinct systems can be employed to assess an agent’s action: one system evaluates the action’s causal aspects, while the other system assesses the agent’s intentions. After assessing the agent’s causal role and intention to cause harm, moral evaluations are conducted to elicit distinct moral responses. When evaluating an agent’s causal and moral responsibility, individuals may judge them based on whether harm was inflicted. For instance, if harm was caused, it could lead to the conclusion that the agent is morally blameworthy. Similarly, if the agent intended to cause harm, they might be perceived as bad based on their intentional moral evaluation. Notably, these two moral evaluations can align (as in the example), clash (when someone causes a negative outcome with a neutral or positive intention) or compete (when someone has a negative intention but yields no harm). The KE represents a specific situation in which the negative outcome conflicts with the neutral intention of the agent. According to both the classical and 2.0 Dual Process models (Kahneman, 2011; De Neys and Pennycook, 2019), negative consequences, generating a strong emotional response, would prompt heuristic responses (System 1). Consequently, this could lead to higher intentionality ratings for the negative side effect, as participants are inclined to blame the agent. The presence of negative elements in an action often leads to a general negative judgment of the agent and an increased attribution of intentionality, even in the case of neutral intentions. In this case, the emotional arousal plays a significant role in interpreting events, as observed in previous studies (e.g., Ngo et al., 2015; Zucchelli et al., 2019). However, a cognitive conflict occurs also when someone unintentionally causes a positive outcome. This conflict arises because the situation does not fit with our typical understanding of someone who intends to do a good deed. As a result, our perception of the person who caused the positive outcome is disrupted (Ngo et al., 2015; Nadelhoffer, 2004; Sripada and Konrath, 2011; Sripada, 2010). In typical unintended consequence situations, the person who performs the action is not interested in the positive outcomes that may result. This can be disconcerting and lead people to believe that the person does not deserve praise for the good that has resulted. This results in a decreased willingness to perceive the side effects as intentional. As we posited in the above interpretation, this disruption of the statistical norms creates a conflict that, following the Dual Process model 2.0, is less strong than the conflict generated by the negative side effect. In this case, we can have a more System 2-like response involving Theory of Mind and Inhibition control (ETIC model) on interpreting the event, which results in a more deliberate response, as shown by slower response times. Consequently, under cognitive load, this more System 2 like response is easily disrupted, resulting in lower intentionality attribution to the positive side effect. According to previous neuroimaging studies (e.g., Ngo et al., 2015), emotional arousal is not involved in the intentionality attribution of the positive side effect. Therefore, according to our results, the ETIC model (Buon et al., 2016) could also be adapted to the intentionality decision-making of the Knobe scenario considering both Dual Process models (see Figure 9) under extraneous cognitive load.

In conclusion, it was important to introduce an e*xtraneous* cognitive load to confirm the presence of two competing responses in the intentionality decision-making of the Knobe scenario. Previous literature (e.g., Martin et al., 2021) has shown that accidental harms are judged more harshly when people, under cognitive load, focus more on causation than on the absence of malicious intent. Similarly, the Knobe Effect demonstrates that a high level of e*xtraneous* cognitive load leads to intuitive responses for positive outcomes, resulting in reduced intentionality attributed to these outcomes, as indicated by slower response times and the intervention of System 2. However, the expected increase in intentionality judgments for negative outcomes was not observed, likely due to the limited variance in data across conditions with and without cognitive load. Despite this, faster response times for negative conditions compared to positive ones confirm the involvement of System 1. This study investigated the role of cognitive load in decision-making regarding intentionality attribution and provided valuable insights into manipulating cognitive load within the context of Dual Process theories. The intrinsic nature of these two types of side effects in the KE scenarios demonstrates how, according to Dual Process Theory 2.0, manipulating cognitive load can be highly effective in situations where there is no strong intuitive bias or false response, as in the case of positive side effects. On the other hand, when we encounter strong intuitive bias or false responses, as for the negative outcomes, a significant cognitive load manipulation is required to elicit an even stronger intuitive response.

### 4.1 Limitations and future research

Our research offers valuable insights into the cognitive models used to understand how people attribute intentionality, but it does have its limitations. We conducted a study comparing the classic and revised Dual Process models using a different experimental procedure from the typical two-response method used by De Neys (e.g., De Neys and Bialek, 2017). In De Neys’ method, commonly employed in studies on probabilistic reasoning, participants are presented with a reasoning problem and asked to give a quick initial response within a time limit. They then have the opportunity to reflect and provide a final answer without a time limit. In contrast, our procedure involved using dual-task and time pressure to deplete cognitive resources, but it did not include an opportunity for participants to reconsider their initial response. Furthermore, we used a between-subject design, while the classical two-response method is a within-subject design, involving the same participant providing two consecutive responses. The approach we chose was influenced by previous experiments on cognitive load and accidental harm (Buon et al., 2013; Martin et al., 2021). In future studies, De Neys’ methodology could be adopted to examine the relationship between KE and moral reasoning in cases of accidental or attempted harm. This could provide valuable insights into the cognitive load, free-thinking, and individual differences in responses. We used two different methods to create cognitive strain — dual-tasking and time pressure — with the goal of depleting cognitive resources. Extensive research indicates that cognitive overload can result in a reliance on automatic or heuristic decision-making processes (Greene et al., 2008; Paxton et al., 2012). This is particularly significant in legal settings, where cognitive strain can affect jurors’ ability to accurately determine fault and appropriate punishment (Kleider-Offutt et al., 2016). In Study 1, the expected impact of cognitive load on negative side effects was not as significant as anticipated. Our decision was based on the work of Bialek and De Neys, which places a heavy demand on participants’ executive resources (De Neys and Verschueren, 2006; Franssens and De Neys, 2009; Miyake et al., 2001). However, the material used in the concurrent task may have influenced the results. The cognitive load hypothesis suggests that high cognitive load can make it more difficult for the brain to ignore irrelevant distractions, leading to increased interference (Kim et al., 2005; Sweller, 1988). The impact of different types of working memory load may vary depending on how they overlap with mechanisms involved in processing the target or distractors or instance, and, in this case, mechanisms for assigning intentionality (verbal format) and resolving dot matrices (visuospatial format) might not overlap sufficiently, allowing participants to compensate by taking more time if there is no adequate time limit. Other studies in moral cognition have used visuospatial scenario materials with a phonologic rather than visual cognitive load, yielding better results for negative outcomes (Buon et al., 2013; Martin et al., 2021). Future studies should consider cognitive loads that align more closely with the mechanisms involved in the intentionality task. Furthermore, using a secondary task that puts pressure on inhibitory control, such as a go-no-go or stop-signal task, may be more effective in reducing the cognitive resources involved in attributing intentionality to negative side effects. Buon et al.’s ETIC model (2016), indeed, emphasizes the role of inhibitory control in suppressing negative emotional arousal. Our combination of a secondary visual load task with a time limit had a noticeable impact, however only on positive side effect; this suggests the opportunity to use different secondary tasks, stressing more inhibitory control, in future studies to reach clearer results also for negative side effects. Schwartz et al. (2024) applied the two-response paradigm to moral scenarios using a within-subject design, revealing that participants were more critical of intentional transgressors and more forgiving of accidental transgressors in their second response when not under cognitive load. These findings support our hypothesis on the positive side effect and highlight the utility of incorporating time constraints in secondary load tasks and utilizing within-subject designs like the two-response paradigm. Despite its limitations, our study introduces varying levels of extraneous cognitive load to understand two Dual Process thinking models in intentionality attribution tasks. The study suggests that the revised Dual Process model may be more reliable than the traditional model in comprehending intuitive and deliberative responses in complex situations, such as the KE scenario.

## Data Availability

The datasets presented in this study can be found in online repositories. The names of the repository/repositories and accession number(s) can be found at: https://osf.io/32p7v/?view_only=908029634f1f40428a2042b2402b7b62.

## Footnotes

^1^Available at: https://www.qualtrics.com.
