# Supplementary material for: The Dual Process model: the effect of cognitive load on the ascription of intentionality
Source: Front Psychol. 2025 Mar 7;16:1451590. doi: 10.3389/fpsyg.2025.1451590 (PMC11927432; doi:10.3389/fpsyg.2025.1451590)
Supplement: Supplementary file 1 [file Supplementary_file_1.docx]

**The Dual Process model: The effect of cognitive load on the ascription of intentionality**

Micaela Maria Zucchelli^1, *^, Nicola Matteucci Armandi Avogli Trotti^1^, Andrea Pavan^1^, Laura Piccardi^2,3^, Raffaella Nori^1,*^

^1^Department of Psychology, University of Bologna, Bologna, Bo, Italy

^2^Department of Psychology, “Sapienza” University of Rome, Rome, RM, Italy

^3^San Raffaele Cassino Hospital, Cassino (FR), Italy

*** Corresponding Authors:**

Dr. Micaela Maria Zucchelli, Ph.D.

Department of Psychology, University of Bologna

V.le Berti Pichat, 5 40127 Bologna, Italy

e-mail: micaela.zucchelli3@unibo.it

Prof. Raffaella Nori, Ph.D.

Department of Psychology, University of Bologna

V.le Berti Pichat, 5 40127 Bologna, Italy

e-mail: raffaella.nori@unibo.it

<https://spaceandvirtualrealitylab.com/>

**Supplementary Material**

1. **Assessing statistical assumptions**

**1.1. Experiment 1**

*1.1.1. Intentionality scores*

The normality of residuals was assessed visually using a QQ plot and the Shapiro-Wilk test. The QQ plot revealed that residuals were not normally distributed for all six groups. The Shapiro-Wilk test was also significant for all groups. The skewness and kurtosis values suggested only a minor departure from normality for some groups (see table S1). Levene’s test for homogeneity of variances was statistically significant (*F*(15, 1544) = 6.87, *p* < 0.001). Three outliers were detected using the boxplot method.

| **Table S1.** Descriptives for intentionality scores. | | | | | | | | | | | | | | | | | | | | | |
| --- | --- | --- | --- | --- | --- | --- | --- | --- | --- | --- | --- | --- | --- | --- | --- | --- | --- | --- | --- | --- | --- |
|  | | | | | | | | | |  | | | |  | | | | **Shapiro-Wilk** | | | |
|  | | **Valence** | | **Load** | | **Mean** | | **SD** | | **Skewness** | | **SE** | | **Kurtosis** | | **SE** | | **W** | | **p** | |
| Intentionality |  | Negative |  | High |  | 5.00 |  | 1.64 |  | -0.6694 |  | 0.152 |  | -0.350 |  | 0.303 |  | 0.907 |  | < .001 |  |
|  |  |  |  | Low |  | 4.99 |  | 1.87 |  | -1.0176 |  | 0.155 |  | 0.253 |  | 0.308 |  | 0.865 |  | < .001 |  |
|  |  |  |  | None |  | 5.12 |  | 1.80 |  | -0.8597 |  | 0.150 |  | -0.129 |  | 0.299 |  | 0.870 |  | < .001 |  |
|  |  | Positive |  | High |  | 2.06 |  | 2.18 |  | 0.8174 |  | 0.148 |  | -0.527 |  | 0.294 |  | 0.844 |  | < .001 |  |
|  |  |  |  | Low |  | 2.11 |  | 2.18 |  | 0.8916 |  | 0.152 |  | -0.285 |  | 0.303 |  | 0.847 |  | < .001 |  |
|  |  |  |  | None |  | 3.30 |  | 2.04 |  | -0.0164 |  | 0.150 |  | -1.052 |  | 0.299 |  | 0.944 |  | < .001 |  |
|  | | | | | | | | | | | | | | | | | | | | | |

- - 1. *Response times*

The normality of residuals was assessed visually using a QQ plot and the Shapiro-Wilk test. The QQ plot showed that residuals were not normally distributed for all six groups. Likewise, the Shapiro-Wilk test was also significant for each of the groups. The skewness and kurtosis values indicated a significant departure from normality for most of the groups (see table S2). Levene’s test for homogeneity of variances was statistically significant (*F*(15, 1544) = 2.74, *p* < 0.001). Multiple outliers were detected using the boxplot method.

| **Table S2.** Descriptives for reaction times (s). | | | | | | | | | | | | | | | | | | | | | | | |
| --- | --- | --- | --- | --- | --- | --- | --- | --- | --- | --- | --- | --- | --- | --- | --- | --- | --- | --- | --- | --- | --- | --- | --- |
|  | | | | | | | | | | |  | | | | |  | | | | **Shapiro-Wilk** | | | |
|  | | **Valence** | | | **Load** | | **Mean** | | **SD** | | **Skewness** | | | **SE** | | **Kurtosis** | | **SE** | | **W** | | **p** | |
| Reaction Times |  | | Negative |  | High |  | 7.63 |  | 5.09 |  | 1.70 |  | 0.155 | |  | 4.67 |  | 0.308 |  | 0.865 |  | < .001 |  |
|  |  | |  |  | Low |  | 4.72 |  | 5.33 |  | 3.49 |  | 0.150 | |  | 16.87 |  | 0.299 |  | 0.630 |  | < .001 |  |
|  |  | |  |  | None |  | 9.50 |  | 10.86 |  | 4.02 |  | 0.148 | |  | 22.15 |  | 0.294 |  | 0.601 |  | < .001 |  |
|  |  | | Positive |  | High |  | 9.89 |  | 9.93 |  | 4.15 |  | 0.152 | |  | 27.69 |  | 0.303 |  | 0.630 |  | < .001 |  |
|  |  | |  |  | Low |  | 5.37 |  | 4.32 |  | 1.92 |  | 0.150 | |  | 4.98 |  | 0.299 |  | 0.830 |  | < .001 |  |
|  |  | |  |  | None |  | 7.63 |  | 5.09 |  | 1.70 |  | 0.155 | |  | 4.67 |  | 0.308 |  | 0.865 |  | < .001 |  |
|  | | | | | | | | | | | | | | | | | | | | | | | |

*1.1.3. Log Response times*

Following the log transformation of reaction times, data were again visually inspected using a QQ plot and the Shapiro-Wilk test. Residuals were not normally distributed for four out of six groups. The skewness and kurtosis values did not suggest a departure from normality for any groups (see Table S3). Levene’s test for homogeneity of variances was not statistically significant (*F*(15, 1544) = 1.22, *p* = 0.247). Twenty outliers were detected using the boxplot method and were included in the analyses.

| **Table S3.** Descriptives for log-transformed reaction times (s). | | | | | | | | | | | | | | | | | | | | | |
| --- | --- | --- | --- | --- | --- | --- | --- | --- | --- | --- | --- | --- | --- | --- | --- | --- | --- | --- | --- | --- | --- |
|  | | | | | | | | | |  | | | |  | | | | **Shapiro-Wilk** | | | |
|  | | **Load** | | **Valence** | | **Mean** | | **SD** | | **Skewness** | | **SE** | | **Kurtosis** | | **SE** | | **W** | | **p** | |
| Log  Reaction  Times |  | High |  | Negative |  | 1.89 |  | 0.730 |  | -0.00415 |  | 0.152 |  | -0.521 |  | 0.303 |  | 0.993 |  | 0.258 |  |
|  |  |  |  | Positive |  | 1.83 |  | 0.640 |  | -0.02302 |  | 0.155 |  | -0.505 |  | 0.308 |  | 0.991 |  | 0.126 |  |
|  |  | Low |  | Negative |  | 1.16 |  | 0.872 |  | -0.05942 |  | 0.150 |  | 1.107 |  | 0.299 |  | 0.983 |  | 0.003 |  |
|  |  |  |  | Positive |  | 1.90 |  | 0.785 |  | 0.56548 |  | 0.148 |  | 0.247 |  | 0.294 |  | 0.977 |  | < .001 |  |
|  |  | None |  | Negative |  | 2.01 |  | 0.701 |  | 0.67764 |  | 0.152 |  | 0.307 |  | 0.303 |  | 0.969 |  | < .001 |  |
|  |  |  |  | Positive |  | 1.36 |  | 0.867 |  | -0.50929 |  | 0.150 |  | -0.184 |  | 0.299 |  | 0.962 |  | < .001 |  |
|  | | | | | | | | | | | | | | | | | | | | | |

**1.2. Experiment 2**

*1.2.1. Intentionality scores*

The QQ plot indicated that residuals were not normally distributed for all six groups. The Shapiro-Wilk test was also significant for all groups. The skewness and kurtosis values suggested only a minor departure from normality for some groups (see table S4). Levene’s test for homogeneity of variances was significant (*F*(5, 1663) = 12.5, *p* < 0.001). No outliers were detected using the boxplot method.

| **Table S4.** Descriptives for intentionality scores. | | | | | | | | | | | | | | | | | | | | | |
| --- | --- | --- | --- | --- | --- | --- | --- | --- | --- | --- | --- | --- | --- | --- | --- | --- | --- | --- | --- | --- | --- |
|  | | | | | | | | | |  | | | |  | | | | **Shapiro-Wilk** | | | |
|  | | **Valence** | | **Load** | | **Mean** | | **SD** | | **Skewness** | | **SE** | | **Kurtosis** | | **SE** | | **W** | | **p** | |
| Intentionality |  | Negative |  | High |  | 5.46 |  | 1.44 |  | -0.889 |  | 0.141 |  | 0.253 |  | 0.281 |  | 0.875 |  | < .001 |  |
|  |  |  |  | Low |  | 5.13 |  | 1.58 |  | -0.719 |  | 0.156 |  | 0.228 |  | 0.310 |  | 0.901 |  | < .001 |  |
|  |  |  |  | None |  | 4.97 |  | 1.79 |  | -0.662 |  | 0.129 |  | -0.358 |  | 0.256 |  | 0.900 |  | < .001 |  |
|  |  | Positive |  | High |  | 1.89 |  | 2.20 |  | 0.995 |  | 0.159 |  | -0.319 |  | 0.317 |  | 0.803 |  | < .001 |  |
|  |  |  |  | Low |  | 1.71 |  | 1.90 |  | 1.026 |  | 0.153 |  | 0.151 |  | 0.306 |  | 0.834 |  | < .001 |  |
|  |  |  |  | None |  | 2.84 |  | 1.97 |  | 0.379 |  | 0.146 |  | -0.671 |  | 0.290 |  | 0.937 |  | < .001 |  |
|  | | | | | | | | | | | | | | | | | | | | | |

*1.2.2. Response times*

The QQ plot showed that residuals were not normally distributed for all six groups. The Shapiro-Wilk test was also significant for each of these groups. The skewness and kurtosis values suggested a significant departure from normality for the two groups without time limit (see Table S5). Levene’s test for homogeneity of variances was statistically significant (*F*(5, 1663) = 42.9, *p* < 0.001). Multiple outliers were detected using the boxplot method.

| **Table S5.** Descriptives for reaction times. | | | | | | | | | | | | | | | | | | | | | |
| --- | --- | --- | --- | --- | --- | --- | --- | --- | --- | --- | --- | --- | --- | --- | --- | --- | --- | --- | --- | --- | --- |
|  | | | | | | | | | |  | | | |  | | | | **Shapiro-Wilk** | | | |
|  | | **Valence** | | **Load** | | **Mean** | | **SD** | | **Skewness** | | **SE** | | **Kurtosis** | | **SE** | | **W** | | **p** | |
| Reaction Times |  | Negative |  | High |  | 2.62 |  | 1.22 |  | 0.357 |  | 0.141 |  | -0.831 |  | 0.281 |  | 0.961 |  | < .001 |  |
|  |  |  |  | Low |  | 2.79 |  | 1.23 |  | 0.272 |  | 0.156 |  | -0.833 |  | 0.310 |  | 0.971 |  | < .001 |  |
|  |  |  |  | None |  | 5.63 |  | 9.59 |  | 5.503 |  | 0.129 |  | 35.190 |  | 0.256 |  | 0.421 |  | < .001 |  |
|  |  | Positive |  | High |  | 3.25 |  | 1.26 |  | 0.127 |  | 0.159 |  | -0.667 |  | 0.317 |  | 0.982 |  | 0.005 |  |
|  |  |  |  | Low |  | 3.09 |  | 1.23 |  | 0.302 |  | 0.153 |  | -0.687 |  | 0.306 |  | 0.971 |  | < .001 |  |
|  |  |  |  | None |  | 5.75 |  | 6.21 |  | 3.072 |  | 0.146 |  | 12.598 |  | 0.290 |  | 0.682 |  | < .001 |  |
|  | | | | | | | | | | | | | | | | | | | | | |

*1.2.3. Log Response Times*

Following the log transformation of reaction times, data were visually inspected using a QQ plot. The residuals were still not normally distributed for all six groups. The skewness and kurtosis values indicate no significant departure from normality (see Table S6). Levene’s test for homogeneity of variances was still statistically significant (*F*(5, 1663) = 50, *p* < 0.001). Thirteen outliers were detected using the boxplot method and were included in the analyses.

| **Table S6.** Descriptives for log-transformed reaction times (s). | | | | | | | | | | | | | | | | | | | | | |
| --- | --- | --- | --- | --- | --- | --- | --- | --- | --- | --- | --- | --- | --- | --- | --- | --- | --- | --- | --- | --- | --- |
|  | | | | | | | | | | **Skewness** | | | | **Kurtosis** | | | | **Shapiro-Wilk** | | | |
|  | | **Valence** | | **Load** | | **Mean** | | **SD** | | **Skewness** | | **SE** | | **Kurtosis** | | **SE** | | **W** | | **p** | |
| Log  Reaction Times |  | Negative |  | High |  | 0.841 |  | 0.518 |  | -0.415 |  | 0.141 |  | -0.714 |  | 0.281 |  | 0.964 |  | < .001 |  |
|  |  |  |  | Low |  | 0.913 |  | 0.499 |  | -0.543 |  | 0.156 |  | -0.473 |  | 0.310 |  | 0.961 |  | < .001 |  |
|  |  |  |  | None |  | 1.215 |  | 0.899 |  | 0.715 |  | 0.129 |  | 0.998 |  | 0.256 |  | 0.968 |  | < .001 |  |
|  |  | Positive |  | High |  | 1.090 |  | 0.454 |  | -0.928 |  | 0.159 |  | 0.886 |  | 0.317 |  | 0.942 |  | < .001 |  |
|  |  |  |  | Low |  | 1.040 |  | 0.432 |  | -0.421 |  | 0.153 |  | -0.667 |  | 0.306 |  | 0.967 |  | < .001 |  |
|  |  |  |  | None |  | 1.360 |  | 0.860 |  | 0.289 |  | 0.146 |  | -0.297 |  | 0.290 |  | 0.988 |  | 0.022 |  |
|  | | | | | | | | | | | | | | | | | | | | | |

**2. Control Analyses for the Influence of Demographic Variables**

**2.1 Experiment 1**

**2.1.1. Analysis of Intentionality Scores and Demographics**

*2.1.1.1. Age*

Model:

int_score ~ load + valence + age + load:valence + age:valence + age:load + age:valence:load + ( 1 | S )

| **Table S7.** Fixed Effects Omnibus Tests | | | | | | | | | |
| --- | --- | --- | --- | --- | --- | --- | --- | --- | --- |
|  | | **F** | | **df** | | **df (res)** | | **p** | |
| load |  | 4.340 |  | 2 |  | 183 |  | 0.014 |  |
| valence |  | 132.346 |  | 1 |  | 183 |  | < .001 |  |
| age |  | 1.271 |  | 1 |  | 183 |  | 0.261 |  |
| load ✻ valence |  | 2.458 |  | 2 |  | 183 |  | 0.088 |  |
| valence ✻ age |  | 0.259 |  | 1 |  | 183 |  | 0.611 |  |
| load ✻ age |  | 1.397 |  | 2 |  | 183 |  | 0.250 |  |
| load ✻ valence ✻ age |  | 0.574 |  | 2 |  | 183 |  | 0.564 |  |
|  | | | | | | | | | |

*2.1.1.2. Education*

Model:

int_score ~ load + valence + education + load:valence + education:valence + education:load + education:valence:load + ( 1 | S )

| **Table S8.** Fixed Effects Omnibus Tests | | | | | | | | | |
| --- | --- | --- | --- | --- | --- | --- | --- | --- | --- |
|  | | **F** | | **df** | | **df (res)** | | **p** | |
| load |  | 3.56473 |  | 2 |  | 183 |  | 0.030 |  |
| valence |  | 128.85687 |  | 1 |  | 183 |  | < .001 |  |
| education |  | 0.23971 |  | 1 |  | 183 |  | 0.625 |  |
| load ✻ valence |  | 2.46927 |  | 2 |  | 183 |  | 0.087 |  |
| load ✻ education |  | 1.19485 |  | 2 |  | 183 |  | 0.305 |  |
| valence ✻ education |  | 0.00402 |  | 1 |  | 183 |  | 0.949 |  |
| load ✻ valence ✻ education |  | 1.09931 |  | 2 |  | 183 |  | 0.335 |  |
|  | | | | | | | | | |

*2.1.1.3. Gender*

Model:

int_score ~ gender + load + valence + gender:load + gender:valence + load:valence + gender:load:valence + ( 1 | S )

| **Table S9.** Fixed Effects Omnibus Tests | | | | | | | | | |
| --- | --- | --- | --- | --- | --- | --- | --- | --- | --- |
|  | | **F** | | **df** | | **df (res)** | | **p** | |
| Gender |  | 0.0358 |  | 1 |  | 183 |  | 0.850 |  |
| Load |  | 4.1268 |  | 2 |  | 183 |  | 0.018 |  |
| Valence |  | 123.0342 |  | 1 |  | 183 |  | < .001 |  |
| gender ✻ load |  | 0.1147 |  | 2 |  | 183 |  | 0.892 |  |
| gender ✻ valence |  | 0.1691 |  | 1 |  | 183 |  | 0.681 |  |
| load ✻ valence |  | 1.9579 |  | 2 |  | 183 |  | 0.144 |  |
| gender ✻ load ✻ valence |  | 0.9120 |  | 2 |  | 183 |  | 0.404 |  |
|  | | | | | | | | | |

**2.1.2. Analysis of Response Times and Demographic Variables**

*2.1.2.1. Age*

Model:

LOGRT ~ load + valence + age + load:valence + age:valence + age:load + age:valence:load + ( 1 | S )

| **Table S10.** Fixed Effects Omnibus Tests | | | | | | | | | |
| --- | --- | --- | --- | --- | --- | --- | --- | --- | --- |
|  | | **F** | | **df** | | **df (res)** | | **p** | |
| load |  | 42.10912 |  | 2 |  | 183 |  | < .001 |  |
| valence |  | 3.92341 |  | 1 |  | 183 |  | 0.049 |  |
| age |  | 0.00523 |  | 1 |  | 183 |  | 0.942 |  |
| load ✻ valence |  | 0.77658 |  | 2 |  | 183 |  | 0.461 |  |
| valence ✻ age |  | 0.37834 |  | 1 |  | 183 |  | 0.539 |  |
| load ✻ age |  | 0.30881 |  | 2 |  | 183 |  | 0.735 |  |
| load ✻ valence ✻ age |  | 0.24593 |  | 2 |  | 183 |  | 0.782 |  |
|  | | | | | | | | | |

*2.1.2.2. Education*

Model: LOGRT ~ load + valence + education + load:valence + education:valence + education:load + education:valence:load + ( 1 | S )

| **Table S11.** Fixed Effects Omnibus Tests | | | | | | | | | |
| --- | --- | --- | --- | --- | --- | --- | --- | --- | --- |
|  | | **F** | | **df** | | **df (res)** | | **p** | |
| load |  | 41.2102 |  | 2 |  | 183 |  | < .001 |  |
| valence |  | 3.7077 |  | 1 |  | 183 |  | 0.056 |  |
| education |  | 0.0144 |  | 1 |  | 183 |  | 0.905 |  |
| load ✻ valence |  | 0.9333 |  | 2 |  | 183 |  | 0.395 |  |
| load ✻ education |  | 0.4869 |  | 2 |  | 183 |  | 0.615 |  |
| valence ✻ education |  | 1.2738 |  | 1 |  | 183 |  | 0.261 |  |
| load ✻ valence ✻ education |  | 0.4154 |  | 2 |  | 183 |  | 0.661 |  |
|  | | | | | | | | | |

*2.1.2.3. Gender*

Model: LOGRT ~ gender + load + valence + gender:load + gender:valence + load:valence + gender:load:valence + ( 1 | S )

| **Table S12** Fixed Effects Omnibus Tests | | | | | | | | | |
| --- | --- | --- | --- | --- | --- | --- | --- | --- | --- |
|  | | **F** | | **df** | | **df (res)** | | **p** | |
| gender |  | 0.557 |  | 1 |  | 183 |  | 0.456 |  |
| load |  | 38.675 |  | 2 |  | 183 |  | < .001 |  |
| valence |  | 2.863 |  | 1 |  | 183 |  | 0.092 |  |
| gender ✻ load |  | 0.806 |  | 2 |  | 183 |  | 0.448 |  |
| gender ✻ valence |  | 1.094 |  | 1 |  | 183 |  | 0.297 |  |
| load ✻ valence |  | 0.463 |  | 2 |  | 183 |  | 0.630 |  |
| gender ✻ load ✻ valence |  | 1.130 |  | 2 |  | 183 |  | 0.325 |  |

**2.2. Experiment 2**

**2.2.1. Analysis of Intentionality Scores and Demographics**

*2.2.1.1. Age*

Model:

int_score ~ load + valence + age + load:valence + age:valence + age:load + age:valence:load + ( 1 | S )

| **Table S13.** Fixed Effects Omnibus Tests | | | | | | | | | |
| --- | --- | --- | --- | --- | --- | --- | --- | --- | --- |
|  | | **F** | | **df** | | **df (res)** | | **p** | |
| Load |  | 2.221 |  | 2 |  | 221 |  | 0.111 |  |
| Valence |  | 275.756 |  | 1 |  | 221 |  | < .001 |  |
| Age |  | 4.03e-5 |  | 1 |  | 222 |  | 0.995 |  |
| load ✻ valence |  | 5.843 |  | 2 |  | 221 |  | 0.003 |  |
| load ✻ age |  | 0.895 |  | 2 |  | 222 |  | 0.410 |  |
| valence ✻ age |  | 2.462 |  | 1 |  | 222 |  | 0.118 |  |
| load ✻ valence ✻ age |  | 0.758 |  | 2 |  | 222 |  | 0.470 |  |
|  | | | | | | | | | |

*2.2.1.2. Education*

Model:

int_score ~ load + valence + education + load:valence + education:valence + education:load + education:valence:load + ( 1 | S )

| **Table S14.** Fixed Effects Omnibus Tests | | | | | | | | | |
| --- | --- | --- | --- | --- | --- | --- | --- | --- | --- |
|  | | **F** | | **df** | | **df (res)** | | **p** | |
| Valence |  | 289.907 |  | 1 |  | 221 |  | < .001 |  |
| Load |  | 2.513 |  | 2 |  | 221 |  | 0.083 |  |
| Education |  | 2.415 |  | 1 |  | 221 |  | 0.122 |  |
| valence ✻ load |  | 6.175 |  | 2 |  | 221 |  | 0.002 |  |
| load ✻ education |  | 1.332 |  | 2 |  | 221 |  | 0.266 |  |
| valence ✻ education |  | 0.437 |  | 1 |  | 221 |  | 0.509 |  |
| valence ✻ load ✻ education |  | 1.762 |  | 2 |  | 221 |  | 0.174 |  |
|  | | | | | | | | | |

*2.2.1.3. Gender*

Model:

int_score ~ gender + load + valence + gender:load + gender:valence + load:valence + gender:load:valence + ( 1 | S )

| **Table S15.** Fixed Effects Omnibus Tests | | | | | | | | | |
| --- | --- | --- | --- | --- | --- | --- | --- | --- | --- |
|  | | **F** | | **df** | | **df (res)** | | **p** | |
| valence |  | 256.099 |  | 1 |  | 221 |  | < .001 |  |
| load |  | 1.884 |  | 2 |  | 221 |  | 0.154 |  |
| gender |  | 0.517 |  | 1 |  | 221 |  | 0.473 |  |
| valence ✻ load |  | 6.084 |  | 2 |  | 221 |  | 0.003 |  |
| valence ✻ gender |  | 2.210 |  | 1 |  | 221 |  | 0.139 |  |
| load ✻ gender |  | 1.010 |  | 2 |  | 221 |  | 0.366 |  |
| valence ✻ load ✻ gender |  | 0.492 |  | 2 |  | 221 |  | 0.612 |  |
|  | | | | | | | | | |

**2.2.2. Analysis of Response Times and Demographic Variables**

*2.2.2.1. Age*

Model:

LOGRT ~ load + valence + age + load:valence + age:valence + age:load + age:valence:load + ( 1 | S )

| **Table S16.** Fixed Effects Omnibus Tests | | | | | | | | | |
| --- | --- | --- | --- | --- | --- | --- | --- | --- | --- |
|  | | **F** | | **df** | | **df (res)** | | **p** | |
| Load |  | 17.4173 |  | 2 |  | 231 |  | < .001 |  |
| Valence |  | 11.6432 |  | 1 |  | 232 |  | < .001 |  |
| Age |  | 0.5268 |  | 1 |  | 235 |  | 0.469 |  |
| load ✻ valence |  | 0.4768 |  | 2 |  | 231 |  | 0.621 |  |
| load ✻ age |  | 0.9741 |  | 2 |  | 234 |  | 0.379 |  |
| valence ✻ age |  | 0.1825 |  | 1 |  | 235 |  | 0.670 |  |
| load ✻ valence ✻ age |  | 0.0349 |  | 2 |  | 234 |  | 0.966 |  |
|  | | | | | | | | | |

*2.2.2.2. Education*

Model: LOGRT ~ load + valence + education + load:valence + education:valence + education:load + education:valence:load + ( 1 | S )

| **Table S17.** Fixed Effects Omnibus Tests | | | | | | | | | |
| --- | --- | --- | --- | --- | --- | --- | --- | --- | --- |
|  | | **F** | | **df** | | **df (res)** | | **p** | |
| Valence |  | 10.2215 |  | 1 |  | 232 |  | 0.002 |  |
| Load |  | 17.0724 |  | 2 |  | 232 |  | < .001 |  |
| Education |  | 0.1499 |  | 1 |  | 233 |  | 0.699 |  |
| valence ✻ load |  | 0.6795 |  | 2 |  | 232 |  | 0.508 |  |
| load ✻ education |  | 1.7997 |  | 2 |  | 232 |  | 0.168 |  |
| valence ✻ education |  | 0.0693 |  | 1 |  | 233 |  | 0.793 |  |
| valence ✻ load ✻ education |  | 0.2758 |  | 2 |  | 232 |  | 0.759 |  |
|  | | | | | | | | | |

*2.2.2.3. Gender*

Model: LOGRT ~ gender + load + valence + gender:load + gender:valence + load:valence + gender:load:valence + ( 1 | S )

| **Table S18.** Fixed Effects Omnibus Tests | | | | | | | | | |
| --- | --- | --- | --- | --- | --- | --- | --- | --- | --- |
|  | | **F** | | **df** | | **df (res)** | | **p** | |
| Valence |  | 8.877 |  | 1 |  | 231 |  | 0.003 |  |
| Load |  | 16.676 |  | 2 |  | 230 |  | < .001 |  |
| Gender |  | 1.257 |  | 1 |  | 231 |  | 0.263 |  |
| valence ✻ load |  | 0.247 |  | 2 |  | 230 |  | 0.781 |  |
| valence ✻ gender |  | 1.170 |  | 1 |  | 231 |  | 0.280 |  |
| load ✻ gender |  | 0.758 |  | 2 |  | 230 |  | 0.470 |  |
| valence ✻ load ✻ gender |  | 0.380 |  | 2 |  | 230 |  | 0.684 |  |
|  | | | | | | | | | |

**3. Time constraint pretest**

The deadline for time constraints was determined based on the average time taken by participants to complete the pretest. Participants were instructed to read and respond as quickly as possible to estimate the minimum time required for task completion. The pretest was conducted with a sample of 31 participants, including 11 males, with an average age of 25.64 and a standard deviation of 3.59 years.

The time constraint for reading the second part of the scenario was set at 6 seconds. This decision was made despite a mean response time of 4.87 seconds (SD = 1.89), as slightly longer response times were observed in scenarios 3 and 4 (see Table S7). This adjustment was made to ensure participants fully understood the scenario. Similarly, the response time constraint for answering the intentionality questions was set at 6 seconds, rounding up from the mean value of 5.66 seconds (SD = 2.80) (see Table S20).

**Table S19.** Time to read the second part of the scenario.

|  | **Mean** | **SE** | **SD** |
| --- | --- | --- | --- |
| Scenario 1 | 4.778 | 0.302 | 1.684 |
| Scenario 2 | 4.787 | 0.382 | 2.129 |
| Scenario 3 | 6.303 | 0.927 | 5.163 |
| Scenario 4 | 6.837 | 0.729 | 4.061 |
| Scenario 5 | 4.157 | 0.186 | 1.035 |
| Scenario 6 | 4.296 | 0.404 | 2.251 |
| Scenario 7 | 4.083 | 0.619 | 3.444 |
| Scenario 8 | 3.744 | 0.448 | 2.493 |
| Scenario total | 4.873 | 0.339 | 1.889 |

**Table S20.** Response time to the intentionality question.

|  | **Mean** | **SE** | **SD** |
| --- | --- | --- | --- |
| Scenario 1 | 5.926 | 0.436 | 2.429 |
| Scenario 2 | 6.602 | 0.624 | 3.472 |
| Scenario 3 | 5.345 | 0.707 | 3.934 |
| Scenario 4 | 6.160 | 0.893 | 4.973 |
| Scenario 5 | 4.830 | 0.504 | 2.808 |
| Scenario 6 | 4.995 | 0.460 | 2.562 |
| Scenario 7 | 5.020 | 0.814 | 4.532 |
| Scenario 8 | 6.437 | 2.050 | 11.416 |
| Scenario total | 5.664 | 0.503 | 2.802 |

1. **Scenarios**

To minimize the amount of information that needed to be read and processed under cognitive load, the scenarios were divided in two parts. The first sentence, which contained general information, was presented before the dot matrix. This division was also preserved in the conditions without cognitive load, ensuring that the cognitive load manipulation was the sole difference between the conditions. The scenarios were adapted from Ngo et al. (2015) and translated into Italian.

**4.1. English version**

**Negative side effect**

1) The farmer spread weed killer to protect her crops.

The farmer did not care about the effect this would have on neighbour’s crops.

The farmer knew his pesticide would also harm neighbour’s 's crops.

Did the farmer intentionally harm his neighbour’s crops?

2) The Director has implemented a new financial plan to increase the company's profits.

The Director did not care about the effect the plan would have on employees.

He knew that the new financial plan would also involve a staff cut.

Did the Director intentionally cut the staff?

3) The reporter released confidential documents about his friend into his article to get it published

on the newspaper's front page.

The reporter did not care about the effect this would have on his friend's reputation. He knew that

disseminating that information would also ruin his friend's reputation.

Did the reporter intentionally ruin his friend's reputation?

4) The Mayor cut spending at the animal shelter to commit them to the maintenance of the public

garden of the city.

The Mayor did not care about the effect the cut would have on the shelter.

He knew that moving funds would also cause the closure of the animal shelter.

Did the Mayor intentionally cause the closure of the animal shelter?

5) The school head has adopted a new meal plan to cut the costs of the canteen. The school head did

not care about the effect this would have on the health of school children. He knew the new meal

plan would also increase the obesity rate among children.

Did the school head intentionally harm the health of the children?

6) The pharmaceutical company has released a new cheaper drug to reduce healthcare costs. The

pharmaceutical company did not care about the effect the new drug would have. They knew the

drug would also increase the rate of vascular disorders.

Has the pharmaceutical company intentionally increased the rate of vascular disease?

7) The CEO started a plan to increase profits.

He did not care about the effect the plan would have on the environment.

He knew her plan would also harm the environment.

Did the CEO intentionally harm the environment?

8) The airplane bomber bombed a factory to reduce enemy’s steel production. He did not care about

the effect the bombing would have on innocent civilians. He knew his bombing would also kill

innocent civilians.

Did the airplane bomber intentionally kill innocent civilians?

**Positive side effect**

1) The farmer spread anti-fungals to protect his crops.

The farmer did not care about the effect this would have on neighbour’s crops. The farmer knew his

anti-fungals would also protect neighbour’s 's crops.

Did the farmer intentionally protect his neighbour’s crops?

2) The Director has implemented a new financial plan to increase the company's profits. The

Director did not care about the effect the plan would have on his employees. He knew the new

financial plan would also lead to new hires.

Did the Director intentionally encourage new hires?

3) The reporter inserted confidential information into his article to get it published on the

newspaper's front page. The reporter did not care about the effect this would have on his Editor-in chief’s reputation. He knew that the dissemination of such information would also give visibility

and notoriety to his Editor-in-chief.

Did the reporter intentionally promote the visibility and notoriety of his Editor-in-chief?

4) The Mayor cut spending at the homeless shelter to engage them in the maintenance of the public

garden. The Mayor did not care about the effect the cut would have on the homeless shelter. He

knew that once the dormitory was out of funds, it would also receive more funds from private

associations.

Did the Mayor intentionally help the dormitory to get more funds from private associations?

5) The school head has adopted a new controlled meal plan to cut canteen costs. The school head

did not care about the effect this would have on children’s health. He knew the new controlled meal

plan would also improve the health of the children.

Did the school head intentionally improve the health of the children?

6) The pharmaceutical company has released a new drug to reduce healthcare costs. The

pharmaceutical company did not care about the effect the new drug would have. They knew the

drug would also decrease the rate of vascular disease.

Has the pharmaceutical company intentionally decreased the rate of vascular disease?

7) The chairman started a plan to increase profits.

He did not care about the effect the plan would have on the environment. He knew his plan would

also help the environment.

Did the chairman intentionally help the environment?

8) The bomber pilot bombed a facility to reduce the enemy’s iron production. He did not care about

the effect the bombing would have on civilians taken hostage. He knew his military action would

also free civilians taken hostage.

Did the bomber pilot intentionally release the civilians taken hostage?

**4.2. Italian adaptation**

**Negative side effect**
1) L’agricoltore ha fatto un trattamento con degli antimicotici per proteggere i propri raccolti. L’agricoltore sapeva che gli antimicotici avrebbero danneggiato anche il raccolto del vicino, ma non gli interessava.

L’agricoltore ha intenzionalmente danneggiato il raccolto del vicino?

2) Il Direttore ha attuato un nuovo piano finanziario per aumentare i profitti dell’azienda.

Il Direttore sapeva che il nuovo piano finanziario avrebbe anche portato ad un taglio del personale, ma non gli interessava.

Il Direttore ha intenzionalmente tagliato il personale?

3) Il giornalista ha inserito nell’articolo delle informazioni confidenziali sul suo amico, per ottenere la pubblicazione nella prima pagina del giornale.

Il giornalista sapeva che la diffusione di quelle informazioni avrebbe anche rovinato la reputazione del suo amico, ma non gli interessava.

Il giornalista ha intenzionalmente favorito la visibilità e notorietà del caporedattore?

4) Il Sindaco ha tagliato i fondi al canile per impegnarli nella manutenzione del giardino pubblico della città.

Il Sindaco sapeva che lo spostamento dei fondi per la manutenzione del giardino pubblico avrebbe anche causato la chiusura del canile, ma non gli interessava.

Il Sindaco ha intenzionalmente causato la chiusura del canile?

5) Il dirigente scolastico ha adottato un nuovo piano alimentare controllato per tagliare i costi della mensa.

Il dirigente scolastico sapeva che il nuovo piano alimentare avrebbe anche aumentato il tasso di obesità tra i bambini, ma non gli interessava.

Il dirigente scolastico ha intenzionalmente danneggiato la salute dei bambini?

6) La casa farmaceutica ha messo in commercio un nuovo farmaco per ridurre i costi sanitari.

Sapeva che il farmaco avrebbe anche aumentato la percentuale di disturbi vascolari, ma non gli interessava.

La casa farmaceutica ha aumentato intenzionalmente la percentuale di disturbi vascolari?

7) Il direttore dell’azienda ha avviato un piano industriale per aumentare i profitti.

Sapeva che il piano industriale avrebbe anche danneggiato l'ambiente ma non gli interessava.

Il direttore ha intenzionalmente danneggiato l'ambiente?

8) Il militare ha colpito una fabbrica per ridurre la produzione di acciaio del nemico.

Sapeva che la sua azione militare avrebbe anche ucciso dei civili, ma non gli interessava.

Il militare ha intenzionalmente ucciso i civili?

**Positive side effect**

1) L’agricoltore ha fatto un trattamento con degli antimicotici per proteggere i propri raccolti. L’agricoltore sapeva che gli antimicotici avrebbero protetto anche il raccolto del vicino, ma non gli interessava.

L’agricoltore ha intenzionalmente protetto il raccolto del vicino?

2) Il Direttore ha attuato un nuovo piano finanziario per aumentare i profitti dell’azienda.

Il Direttore sapeva che il nuovo piano finanziario avrebbe anche portato a nuove assunzioni, ma non gli interessava.

Il Direttore ha intenzionalmente favorito le nuove assunzioni?

3) Il giornalista ha inserito delle informazioni confidenziali nel suo articolo, per ottenere una pubblicazione sulla prima pagina del giornale.

Il giornalista sapeva che la diffusione di tali informazioni avrebbe anche dato visibilità e notorietà al proprio caporedattore, ma non gli interessava.

Il giornalista ha intenzionalmente favorito la visibilità e notorietà del caporedattore?

4) Il Sindaco ha tagliato i fondi al dormitorio per impegnarli nella manutenzione del giardino pubblico.

Il Sindaco sapeva che una volta sprovvisto di fondi, il dormitorio avrebbe ricevuto maggiori fondi da parte delle associazioni private, ma non gli interessava.

Il Sindaco ha intenzionalmente aiutato il dormitorio ad ottenere maggiori fondi da parte delle associazioni private?

5) Il dirigente scolastico ha adottato un nuovo piano alimentare controllato per tagliare i costi della mensa.

Il dirigente scolastico sapeva che il nuovo piano alimentare avrebbe anche migliorato la salute dei bambini, ma non gli interessava.

Il dirigente scolastico ha intenzionalmente migliorato la salute dei bambini?

6) La casa farmaceutica ha messo in commercio un nuovo farmaco per ridurre i costi sanitari.

Sapeva che il farmaco avrebbe anche diminuito la percentuale di disturbi vascolari, ma non gli interessava.

La casa farmaceutica ha intenzionalmente diminuito la percentuale di disturbi vascolari?

7) Il direttore dell’azienda ha avviato un piano industriale per aumentare i profitti.

Sapeva che il piano industriale avrebbe anche aiutato l'ambiente ma non gli interessava.

Il direttore ha intenzionalmente aiutato l'ambiente?

8) Il militare ha colpito una fabbrica per ridurre la produzione di acciaio del nemico.

Sapeva che la sua azione militare avrebbe anche liberato dei civili presi in ostaggio, ma non gli interessava.

Il militare ha intenzionalmente liberato i civili presi in ostaggio?
